# Living Gluten-Free in Romania: A National Cross-Sectional Study of Dietary Adherence in Clinically Diagnosed and Self-Reported Cases

**DOI:** 10.3390/nu17233664

**Published:** 2025-11-23

**Authors:** Dana Stanciu, Hristian Staykov, Stela Dragomanova, Lyubka Tancheva, Simeonka Dimitrova, Emanuel Țundrea, Gianina Crișan

**Affiliations:** 1 Department of Pharmaceutical Botany, Faculty of Pharmacy, “Iuliu Hațieganu” University of Medicine and Pharmacy, 400337 Cluj-Napoca, Romania; gcrisan@umfcluj.ro; 2 Department of Pharmacology and Toxicology, Faculty of Medicine, Medical University of Sofia, 1431 Sofia, Bulgaria; 3 Department of Pharmacology, Toxicology and Pharmacotherapy, Faculty of Pharmacy, Medical University of Varna, 9000 Varna, Bulgaria; stela_dragomanova@abv.bg (S.D.); simeonka.dimitrova@mu-varna.bg (S.D.); 4 Institute of Neurobiology, Bulgarian Academy of Sciences, 1113 Sofia, Bulgaria; lyubkatancheva@gmail.com; 5 Department of Business Informatics, Faculty of Management, “Emanuel” University, 410597 Oradea, Romania; emanuel@emanuel.ro

**Keywords:** glutens, gluten-related disorders, celiac disease, non-celiac gluten sensitivity, gluten-free diet, therapy adherence, dietary management, food labeling, food security, quality of life

## Abstract

**Background/Objectives**: A gluten-free diet (GFD) remains the only effective therapy for celiac disease (CD) and non-celiac gluten sensitivity (NCGS). Strict adherence is essential, yet it can impose considerable psychological, social, and financial burdens. This study investigated factors influencing GFD adherence, explored the perceived burden of this therapy, and examined differences between individuals with CD and NCGS. **Methods**: A cross-sectional anonymous questionnaire was completed by 681 Romanian citizens living in Romania with either a medically confirmed or a self-reported diagnosis of CD or NCGS. The survey assessed GFD adherence and its potential predictors, including gender, family history, comorbidities, diagnostic confirmation, and food security and perceived availability, as well as various psychological, social, and financial factors. **Results**: Participants with CD showed significantly higher GFD adherence than those with NCGS. Self-diagnosis was more common among NCGS respondents and was associated with poorer adherence, whereas a medically confirmed diagnosis predicted stricter adherence. Longer time since diagnosis, a greater perceived importance of a GFD, consistent label reading, as well as weight gain after starting a GFD were also positively associated with adherence. Although gluten-free (GF) food security has improved over time, cost remains a major barrier. Social activities negatively influenced adherence, reflecting the isolating effects of dietary restrictions. Nearly 25% of respondents reported a family history of gluten-related disorders (GRDs). Women—although more frequently affected by GRDs—exhibited levels of adherence similar to men. Comorbidities were common (33.9%), predominantly autoimmune diseases (56.3%), with autoimmune thyroiditis (32%) and lactose intolerance (19.2%) being the most frequent comorbidities. **Conclusions**: Diagnostic certainty, motivation, and practical barriers influence GFD adherence. Enhanced public awareness, clear labeling, improved GF food security, and financial support could facilitate sustained adherence and reduce psychosocial burden. To the author’s knowledge, this is the first national study of its kind in Romania.

## 1. Introduction

Over the past decade, gluten and its related prolamins from wheat, barley, and rye have been intensively discussed as their consumption has been linked to several medical conditions called gluten-related disorders (GRDs). Celiac disease (CD), wheat allergy (WA), and non-celiac gluten sensitivity (NCGS) represent the main GRDs, and they are immune-mediated conditions [1]. GRDs have progressively become an epidemiologically significant occurrence, with an estimated global prevalence of around 5%. As a result, individuals with these disorders must eliminate gluten from their diets and adhere to a gluten-free diet (GFD). The term GRDs is an umbrella designation [2], and in this article, we utilize it to denote both CD and NCGS.

CD is defined as a chronic, multi-organ autoimmune disorder that primarily affects the small intestine in genetically predisposed individuals [3,4]. Its characteristic intestinal manifestations include villous atrophy, crypt hyperplasia, inflammation, and nutrient malabsorption, all of which can affect overall health [5,6]. The most common form of nutrient malabsorption is lactose malabsorption, which often leads to lactose intolerance (LI), since damage to the intestinal lining results in the loss of lactase enzymes located on the intestinal villi [7]. CD patients have the class-II human leukocyte antigen (HLA) DQ2 or DQ8 (and rarely HLA-DQ7) haplotype [5,8,9,10]. Disease pathogenesis includes autoantibodies against tissue transglutaminase type 2 (anti-tTG2), endomysium (anti-EMA), and deamidated gliadin peptides (anti-DGP), all of which are targeted when serological testing is performed for diagnosis [11,12]. Moreover, the diagnosis is confirmed by a combination of clinical assessment, serological tests, and histopathological analysis (small-bowel biopsy) [3]. Understanding CD in childhood is further aided by recent research from Romania. Strong concordance between anti-tTG IgA titers and EMA positivity, frequent HLA-DQ2/DQ8 presence, and a high prevalence of vitamin D deficiencies were found in a large 2025 cohort study of Romanian children referred for CD testing, highlighting the necessity of thorough diagnostic and nutritional evaluation in this population [13].

CD affects approximately 1% of the general population, with a notable rise in the incidence of CD over time, indicating that environmental variables and better diagnostic procedures might be involved [14]. A cross-sectional study of 713 adult participants in Romania revealed a verified prevalence of CD of around 1.3%, with ~67% of the subjects being female, undermining the predominance of women with this diagnosis [15]. Geographical location, age, and sex all affect the incidence of CD, and over time, since 1991, CD has become more common and frequent. It is believed that many cases are still undiagnosed [4,16]. According to Rouvroye et al., for every diagnosed case, there are up to two others that remain undetected [17]. CD is among the most prevalent autoimmune conditions, and the only known treatment available is following a strict lifelong GFD (where even trace gluten contaminants are prohibited) [6,18,19] since it is triggered by exposure to gluten and gluten-like proteins [8,18].

Numerous gastrointestinal and extraintestinal manifestations can be indicative of CD. The diagnosis may become complex due to the varied manifestations of symptoms, as some individuals might display only mild and nonspecific symptoms or could be entirely asymptomatic [20]. In their recent single-center study, Romanian researchers also highlighted the systemic nature of CD. The biopsy-confirmed CD patients frequently presented with extraintestinal manifestations, including iron deficiency anemia (20.37%), Hashimoto’s disease (14.81%), cardiovascular diseases (12.04%), and lupus (11.11%), underscoring the condition’s broad clinical spectrum in this population [21]. Although research on GRDs in Romania is still scarce, available evidence underscores meaningful clinical heterogeneity. A 2016 study assessing the phenotype of clinically diagnosed Romanian adults and children found significant age-related differences in symptoms, antibody levels, and mucosal injury, pointing to important diagnostic challenges in the local population [22].

A presentation of the clinical indicators, symptoms, and other disorders linked to CD is shown in Table 1. Within hours after gluten exposure, patients with CD may experience the usual upper gastrointestinal tract symptoms, including nausea, abdominal discomfort, and systemic signs of immunological activation [23].

NCGS is a complex and poorly understood clinical disorder that presents a variety of gastrointestinal and extraintestinal symptoms, which can occur within hours or days following the consumption of gluten-containing (GC) foods. These symptoms generally subside upon the removal of gluten from the diet, and this condition occurs in the absence of CD or WA (which is IgE-mediated) [3,31]. Although NCGS is categorized as a GRD, it has been suggested that fermentable oligo-, di-, monosaccharides, and polyols (FODMAPs), rather than gluten, might be the primary triggers responsible for the emergence of symptoms. Moreover, most cereals naturally contain amylase trypsin inhibitors (ATIs), which may also exacerbate the clinical manifestations of NCGS [32,33]. Unlike CD and WA, NCGS is a non-autoimmune, non-allergic phenomenon and does not involve autoimmune enteropathy, villous atrophy, or specific serological markers, making its diagnosis challenging. The condition is mainly recognized by the appearance of symptoms after gluten intake and the subsequent improvement on a GFD, frequently depending on the elimination of other disorders [34,35]. Up to 56% of patients with NCGS exhibit high titers of anti-gliadin antibodies (AGA-IgG), indicating their potential utility in clinical practice [36]. Currently, there are no definitive biomarkers for NCGS that have been identified [37]. Future studies should investigate the NCGS genetic basis, histological features, susceptibility, and associated risks in addition to developing trustworthy biomarkers. Moreover, due to symptom overlapping with irritable bowel syndrome (IBS) and CD, and without clear markers, misdiagnosis may be a very common occurrence [3,38,39]. The occurrence of NCGS exhibits significant variability, likely attributable to the absence of clear diagnostic standards. According to the research conducted by Shahbazkhani et al., the prevalence of NCGS among Western populations ranges from 0.6% to 10.6% [36]. Table 2 summarizes the key gastrointestinal and extraintestinal manifestations of NCGS, alongside associated conditions, providing a comprehensive overview of this enigmatic condition.

The characterizations of CD and NCGS highlight both their comparable clinical manifestations and their distinguishing features. It is important to note that NCGS represents a non-autoimmune, non-allergic reaction to gluten and related prolamins, lacking the specific small-bowel injury that is characteristic of CD. In contrast, CD is classified as an autoimmune disorder, marked by specific biomarkers and considerable damage to the intestinal lining. At present, the GFD remains the primary therapy for both conditions, and it can influence patients’ lives in multiple ways. Nevertheless, advances in understanding CD pathogenesis have led to promising therapeutic candidates that, in the future, may complement—or potentially even serve as alternatives to—a GFD [45]. These include glutenases (latiglutenase, zamaglutenase) [46,47], intraluminal gluten-binding agents (P(HEMA-co-SS), scFv, AGY) [48,49,50], zonulin antagonists (lazotide) [51], intestinal tissue transglutaminase inhibitors (ZED1227/TAK-227, si-RNA therapy) [52,53], tolerance-inducing and immune-modulating strategies (DONQ52, TAK-101, KAN-101, cathepsin S inhibitors, anticytokine agents) [54,55,56,57,58], among others [54,59]. Several of these candidates have shown promising early results.

The main objective of this study was to evaluate the effects of the GFD and to analyze the dietary adherence and management of Romanian individuals with either self-diagnosed or medically diagnosed GRDs in relation to this dietary intervention, thereby providing an in-depth insight into the difficulties encountered while adhering to a GFD. Moreover, we sought to determine whether there are any differences between the CD and NCGS groups in terms of adherence, sources of information, associated chronic diseases, heredity, perception of the diet, and other demographic and behavioral characteristics, bringing forth many novel insights. While much research has focused on the medical aspects of GRDs, few studies have explored the psychosocial, economic, and behavioral barriers that influence adherence to a GFD. Furthermore, the effects of social stigma and discrimination on dietary adherence are unclear, and we wanted to navigate such aspects as well. Furthermore, given that LI is frequently associated with GRDs, we aimed to examine its occurrence as a related condition. Self-diagnosis is a practice that warrants significant discouragement, and our objective was to ascertain whether this behavior is prevalent in Romania in relation to GRDs.

To our knowledge, this original study is the first one to provide a thorough analysis of dietary adherence among Romanians with GRDs and to paint a comprehensive picture of the burdens and struggles that this diet presents for this demographic. This research explores under-addressed concerns affecting dietary adherence. The findings of this study are important as they can guide clinical practice by assisting medical practitioners in creating tailored treatments to promote dietary adherence. Moreover, recognizing socioeconomic hurdles may result in legislative reforms that increase public knowledge, improve access to gluten-free (GF) products, and promote improved labeling regulations and patient financial assistance. The Romanian context offers a distinctive setting for this research, characterized by growing recognition of GRDs but persistent disparities in medical awareness, dietary counseling availability, and the affordability of GF products.

## 2. Materials and Methods

### 2.1. Participants

We conducted a questionnaire-based cross-sectional study, and the data were collected during the period from June 2024 to August 2024. Romanian citizens following a GFD were invited to participate in this study through social media platforms and designated online support groups with GRD patients. In total, 724 individuals completed an anonymous online survey. Participants were informed about the present study and that they were participating in this research voluntarily, and with no remuneration. The feedback provided by minors, who are undergoing these circumstances, was given by their legal guardians.

Several inclusion and exclusion criteria were applied. Eligible participants were required to (1) be capable of providing informed consent, (2) be able to complete the questionnaire, (3) report a GRD (specifically CD or NCGS), and (4) hold Romanian citizenship. Individuals were excluded if they (1) declined to provide informed consent, (2) did not report a GRD, (3) were not Romanian nationals, or (4) indicated residence outside Romania at the time of survey completion. The latter two criteria ensured that the study population consisted of Romanian nationals living in Romania. After applying these criteria, 681 participants were included in the final analysis.

### 2.2. Ethical Aspects

The study was conducted in accordance with the Declaration of Helsinki and approved by the Ethics Committee of “Iuliu Hațieganu” University of Medicine and Pharmacy from Cluj-Napoca, Romania (ethical approval number AVZ 235/20 SEP.2023, and the approved amendment AVZ 105/29 APR.2025).

### 2.3. Questionnaire Components

The purpose of the questionnaire was to gather, in an organized yet thorough manner, the lived experiences of people with GRDs, namely NCGS and CD, associated or not with LI (often comorbid with these disorders). Our main goal was to comprehend the participants’ clinical and demographic characteristics as well as their perceptions, adjustments, needs, and coping mechanisms regarding following a lifetime GFD.

The original questionnaire used for this study was in Romanian (available in English as Supplementary Questionnaire S1). It contained thirty-three questions divided into six categories, the last being a testimonial open section where responders could share their experience in more detail, to give voice to personal journeys and to glean qualitative insights. The first five main categories were (a) the informed consent for inclusion in the study (item 1); (b) general patient data and the identification of their pathologies; (c) information about the patient’s diet; (d) the patient’s adherence to diet therapy; and (e) the socioeconomic impact of the patient’s pathology and diet. To increase clarity and simplicity of completion, we used straightforward, single- or multiple-choice or true/false items for each concept. The main concepts are grouped as follows:

1. Residence and gender (items 2–3): to identify the Romanian citizens who continue to reside in Romania and to help us determine the male-to-female ratio for these disorders.
2. Diagnostic background, familial incidence, and comorbidities (items 4–11): to explore how diagnoses are made and to identify self-diagnostic practices, the hereditary factor, emotional adaptation to the diagnosis, and coexistent chronic diseases.
3. Dietary knowledge and attitudes (items 12–16): sources of information and shifting perceptions of importance and difficulty.
4. Accessibility of GF foods (items 17–20): frequency of finding suitable products in stores and restaurants, both at the time of diagnosis and at the time of completing the questionnaire.
5. Physical and social consequences (items 21–23): weight changes, feelings of discrimination, and social absences due to dietary restrictions.
6. Economic burden (item 24): perceived expense of GF alternatives.
7. Adherence behaviors and beliefs (items 25–33): self-reported dietary adherence, label-reading habits, trust in certified products, and strategies in social contexts.

In formulating the questions, we examined the existing scientific literature on GRDs to identify key topics and gaps that would aid in the development of the questionnaire. These include the psychological impacts of this necessarily restrictive therapy; family medical history and diagnostic processes; emotional acceptance; sources of information regarding the GFD; the significance and challenges associated with the diet; the accessibility of GF options in both dining and retail environments; physical outcomes such as changes in weight; experiences of discrimination; levels of social engagement; financial challenges; and adherence behaviors, including label-checking and intentional lapses. To encourage detailed personal stories that would enhance the study’s findings, we included the “bonus” open-ended section. The questionnaire gives us a nuanced picture of life on a GFD by combining quantitative scales with an open narrative prompt that enables us to juxtapose numerical trends with personal stories. A few selected quotes are included in this paper to illustrate relevant quantitative findings, while a future separate analysis will comprehensively explore the full set of qualitative responses.

### 2.4. Adherence with the GFD

To operationalize adherence to a GFD, a composite adherence score was developed. This score was derived by summing the individual responses to a series of questionnaire items specifically designed to evaluate dietary habits and self-reported instances of dietary transgressions. Each item was scored based on predefined criteria that reflect the degree of adherence, with higher scores indicating greater adherence (one item was reverse-coded for this purpose). Some examples of items used to compute the GFD adherence score are: “I follow the gluten-free diet”, “There are situations in which I voluntarily consume gluten”. The summation of these item scores provided an overall measure of GFD adherence, which served as the primary metric for analysis in this study.

### 2.5. Study Hypotheses and Analyses

For this study, a series of hypotheses were formulated and organized into three distinct categories: (1) medical and individual factors, (2) psychosocial factors, and (3) economic and availability factors. This categorization enabled a structured examination of the multifaceted influences on GFD adherence, facilitating targeted analysis across these thematic domains. The proposed hypotheses are listed below (Table 3), along with the auxiliary analyses and study questions (Table 4).

### 2.6. Statistical Analysis

Statistical analyses were performed using JASP version 0.19. A range of analyses was conducted to test the formulated hypotheses, including descriptive statistics, regression analyses, ANOVA, and chi-square tests. These methods were employed to explore associations, identify predictors, and evaluate differences across categories of patients, ensuring a comprehensive assessment of factors influencing GFD adherence.

## 3. Results

### 3.1. Descriptive Statistics and Individual Differences

In this section, we provide sample descriptive statistics for adherence to the GFD measurement, as well as for the variables pertaining to the medical and individual factors, the psychosocial factors, and the economic and accessibility factors. We also present here the results of testing several individual differences with respect to these factors.

On average, males and females tend to exhibit very similar levels of adherence to the GFD, with females showing somewhat greater variability compared to males, as shown in Table 5.

As seen in Table 6, almost 80% of the respondents were diagnosed with CD, while approximately 21% were diagnosed with NCGS. The male-to-female ratio is 1:5.36 across diagnosis, and when distributed by diagnosis, M:F = 1:5.34 for CD and M:F = 1:5.45 for NCGS. We tested whether these diagnoses are related to the presence of other family members with GRDs; we found that, overall, the percentage of patients who reported that they have family members with the same diagnosis is 22.6%. We tested this for statistical significance and found that there is a statistically significant association between the type of diagnosis (NCGS vs. CD) and the presence of other family members with the same diagnosis [χ^2^ (1) = 4.017, *p* = 0.045]. Specifically, a larger percentage of NCGS patients than expected by chance have other family members with the same diagnosis (28.9%), while fewer CD patients than expected by chance have other family members with the same diagnosis (20.97%).

As anticipated, the majority of patients (89%) received their diagnosis from a specialist physician, while merely 11% of patients diagnosed themselves (Table 7). Interestingly, NCGS patients are more likely to self-diagnose compared to CD patients, and this difference is statistically significant [χ^2^ (1) = 178.664, *p* < 0.001]. Thus, among the self-diagnosed patients, 80% are NCGS patients and 20% are CD patients.

### 3.2. Impact of Medical and Individual Factors on GFD Adherence

We tested these hypotheses using the method of linear regression, with GFD adherence as the outcome variable and several variables as predictors. The results of the global test are shown below (see Table 8).

The regression model that includes medical and individual factors is statistically significant at the 5% significance level, as shown in Table 8. Table 9 shows which of these factors are significantly associated with GFD adherence.

Among the various medical and personal factors, the nature of the diagnosis (NCGS versus CD) and whether patients obtained an official diagnosis or opted for self-diagnosis are significant determinants of adherence to a GFD. When controlling for the effect of other variables, the CD patients are more adherent to the GFD compared to the NCGS patients, and the difference in adherence between the two groups of patients is very large, according to Cohen (1988) [60]. This result supports **H2**.

**Figure 1:**
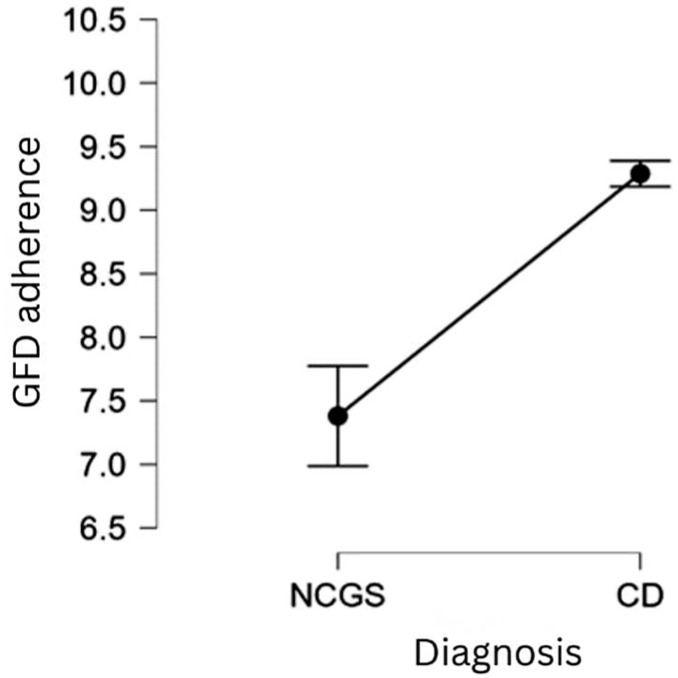
Effect of diagnosis on GFD adherence (GFD = gluten-free diet; CD = celiac disease; NCGS = non-celiac gluten sensitivity).

Figure 1 illustrates the effect of the type of diagnosis on GFD adherence: the level of adherence of CD patients increases by 1.167 units, on average, compared to that of NCGS patients. This is a very large effect (*Cohen’s d* = 1.257) [60].

We took a closer look at the relationship between the type of diagnosis and a particular aspect of GFD adherence: whether or not patients bring their own food to social events (behavior that could foster a more strictly followed GFD). We found that, in fact, significantly more CD patients (91.28%) bring their own food to social events compared to the NCGS patients (77.46%). This difference is statistically significant [χ^2^ (1) = 20.918, *p* < 0.001].

When controlling for the effects of other variables, self-diagnosed patients are significantly less inclined to follow the GFD diet, in support of **H5**. The adherence level of self-diagnosed patients decreased by 0.603 units, on average, compared to the patients who received an official diagnosis. According to Cohen (1988) [60], this is a very large effect (*d* = −1.063).

The remaining variables included in the model were not significantly associated with GFD adherence: we found no effect of having other family members with the same diagnosis, of having other chronic conditions, or of the time passed since the diagnosis. Thus, our data do not support **H1**, **H3**, and **H4**.

We tested whether patients who read the food labels tended to be more GFD adherent, or whether there is an interaction effect between reading the food label and time since diagnosis, on GFD adherence. For this, we conducted a 2-way ANOVA with an interaction effect. We did not find a significant interaction effect [*F*(4,671) = 1.232, *p* = 0.296]. However, we found a very strong effect of reading the food labels on GFD adherence [*F*(1,671) = 86.046, *p* < 0.001, *Cohen’s d* = −2.671]. Thus, patients who often read food labels are significantly more likely to be adherent to the GFD diet compared to those who do not read the labels. This finding supports **H6**.

Interestingly, in both the NCGS and the CD patients, the vast majority regularly check the food product labels. Nonetheless, significantly more CD patients check food product labels compared to the NCGS patients [χ^2^ (1) = 16.339, *p* < 0.001]. Specifically, among the CD patients, approximately 99% responded that they check food product labels, and among the NCGS patients, approximately 94% responded that they check food product labels. Unfortunately, 26.6% of participants stated that they ate foods that were certified as being GF and then had symptoms that proved the certification was untrue (i.e., it contained gluten or traces of gluten that caused specific manifestations to GRDs). This highlights the need for accurate food labelling and certification.

Additionally, we looked at whether there is an association between the patients’ weight fluctuations after their diagnosis and starting the GFD, and GFD adherence. We found that this association is statistically significant [*F*(2,678) = 13.652, *p* < 0.001]. Specifically, the patients whose weight has increased since they started the GFD are more adherent to the GFD compared to those whose weight has decreased [*t*(678) = 4.686, *p* < 0.001, *d* = 0.441]. In addition, the patients whose weight has increased since they started the GFD are more adherent to the GFD compared to those whose weight has remained the same [*t*(678) = 3.955, *p* < 0.001, *d* = 0.361]. These are medium effects according to Cohen (1988) [60].

### 3.3. Impact of Psychosocial Factors on GFD Adherence

To delve deeper into the factors influencing adherence to a GFD, a multiple regression analysis was performed. The model included five key predictors: “difficulty accepting the diagnosis,” “perceived difficulty of the gluten-free diet,” “feeling discriminated against due to diagnosis,” “perceived importance of the gluten-free diet,” and “absence from social contexts.” These variables were selected based on their theoretical relevance and empirical support in influencing dietary adherence behaviors. The outcome variable for the regression model was the composite adherence score, allowing for the assessment of how these psychosocial factors contributed to variations in adherence to the GFD.

The model explains 33.5% of the variance in GFD adherence (see Table 10 below), which is a substantial proportion, given the behavioral nature of the outcome. This model is statistically significant [*F*(20,660) = 16.650, *p* < 0.001], indicating that at least one of the predictors significantly contributes to explaining adherence levels. Table 11 shows the statistically significant predictors. Individuals who perceive the GFD as only “a little important” at the time of the diagnosis, or “(moderately) important” at the time of survey completion, show significantly lower adherence. This suggests that perception of importance is crucial in maintaining dietary restrictions.

**Figure 2:**
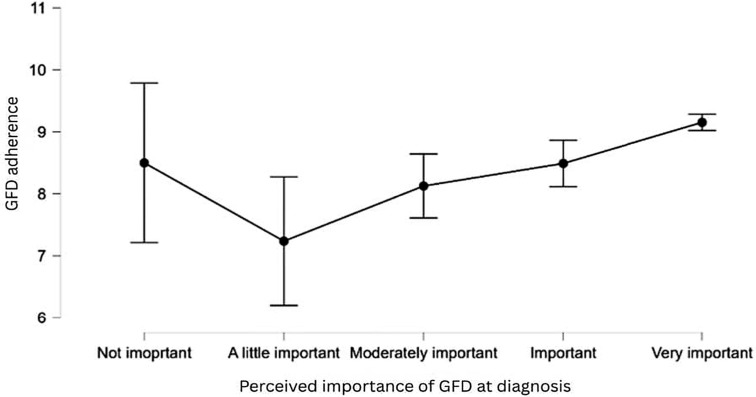
GFD adherence across levels of perceived GFD importance at diagnosis time (GFD = gluten-free diet).

Figure 2 shows that GFD adherence tends to slightly increase with increasing levels of perceived GFD importance, except for those patients who find the GFD “a little important”, who are significantly less adherent to the GFD.

**Figure 3:**
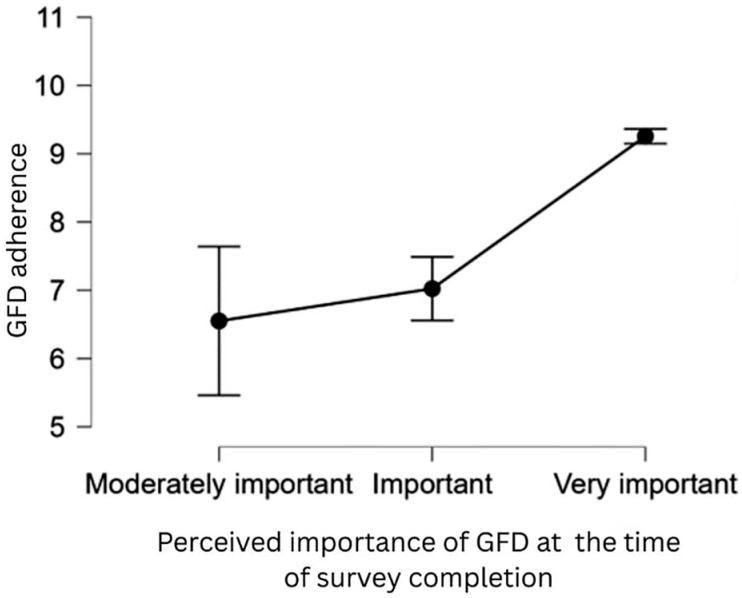
GFD adherence across levels of perceived GFD importance at the time of survey completion (GFD = gluten-free diet).

Moreover, we compared these findings with the data from the moment of survey assessment (Figure 3), and we found that a higher perceived importance of the GFD is associated with increased adherence. These findings support **H9**. With the least variation, participants who ranked the GFD as “very important” at the time of assessment had the greatest levels of adherence. This suggests that adherence is more significantly affected by current motivation or perception rather than by past beliefs. A notable shift in perspective regarding the recognition of the essential role of dietary adherence is evidenced by the substantial decrease in the proportion of individuals who rated the GFD as “not important” or “somewhat important” at the time of completing the questionnaire (0.4%). This shift most likely reflects the impact of symptom management, lived experience, and perhaps more exposure to peer support or instructional materials. The analysis of the two distinct periods suggests that improved adherence could stem from a gradual shift in the understanding of the significance of the GFD, which appears to increase over time. This emphasizes the possible advantages of continuing education and reinforcement in addition to the information provided or available at diagnosis.

**Figure 4:**
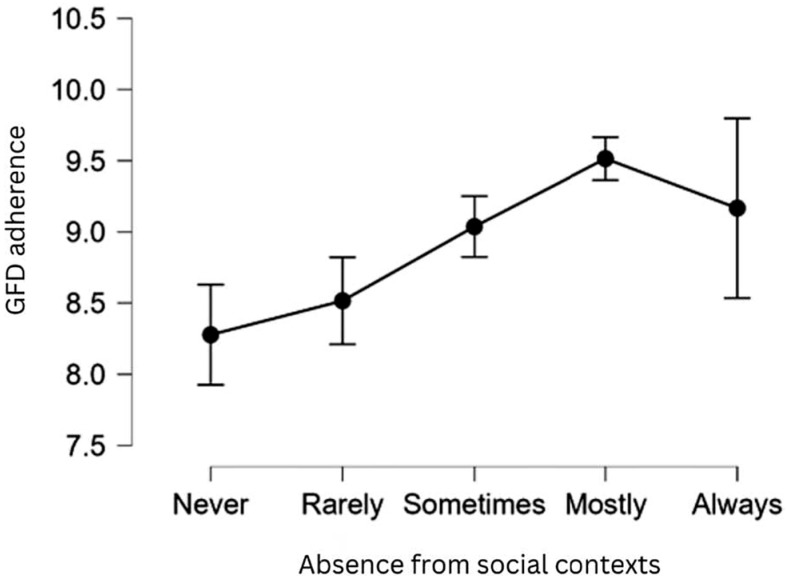
GFD adherence correlated with absence from social contexts (GFD = gluten-free diet).

We found a positive and statistically significant association between the absence from social contexts and GFD adherence: individuals who report being sometimes or mostly absent from social contexts tend to have higher adherence to the GFD. This could suggest that avoiding social situations where gluten exposure is more likely may facilitate better dietary adherence (see Figure 4 below). On the other hand, one might contend that patients who adhere more closely to the GFD often exhibit a tendency to isolate themselves largely. This finding supports **H10**.

The perception of the difficulty of the GFD (both short-term and long-term), feeling discriminated against, and various levels of difficulty in accepting the diagnosis did not show significant contributions individually to adherence levels. Thus, our data do not support **H7**, **H8**, and **H11**. However, we uncovered a number of fascinating additional findings concerning certain variables, which we discuss in detail below.

Among the CD and NCGS patients, most (82.5% of all participants) found the GFD to be difficult at first (at the time of diagnosis). Significantly more CD patients (84%) rated the GFD as “difficult” at first compared to the NCGS patients (76%), and significantly fewer CD patients (0.7%) rated it as “easy” compared to the NCGS patients (2.8%): [χ^2^ (2) = 7.593, *p* = 0.022]. At the time of survey completion, only 20.4% of all participants rated the GFD as “difficult”, 65% rated it as “acceptable”, and these patients were distributed similarly across the two groups. A difference in proportions was found between the CD and NCGS groups, in the sense that significantly fewer CD participants (11.9%) rated the GFD as “Easy” compared to the NCGS participants (23.2%): [χ^2^ (2) = 11.886, *p* = 0.003].

Regarding feelings of discrimination, 65.6% of all participants responded that they feel discriminated against because of their condition. Among these, significantly more are “sometimes” (34.2%), “mostly” (32%), or “always” (4.9%) absent from social events [χ^2^ (4) = 127.575, *p* < 0.001]. This suggests that the patients who feel discriminated against because of their condition tend to isolate themselves more. Moreover, we also found that significantly more CD patients are sometimes or mostly absent from social contexts, compared to the NCGS patients [χ^2^ (4) = 27.954, *p* < 0.001].

### 3.4. Impact of Economic Factors and Availability on GFD Adherence

Ultimately, to delve deeper into the factors influencing adherence with a GFD, we performed another multiple regression analysis. This model incorporated three primary predictors: the perceived financial burden of GF products, the availability of GF options in stores and on restaurant menus at the time of diagnosis, as well as the availability of GF alternatives in stores and on restaurant menus when the questionnaire was completed. The outcome variable for the regression model was the composite adherence score, allowing for the assessment of how these economic and availability factors contributed to variations in adherence to the GFD.

This model is statistically significant at the 5% level (see Table 12 below), but it only explains 5.8% of the variance in GFD adherence, which is a rather modest proportion. Nevertheless, at least one of the predictors significantly contributes to explaining adherence levels.

**Figure 5:**
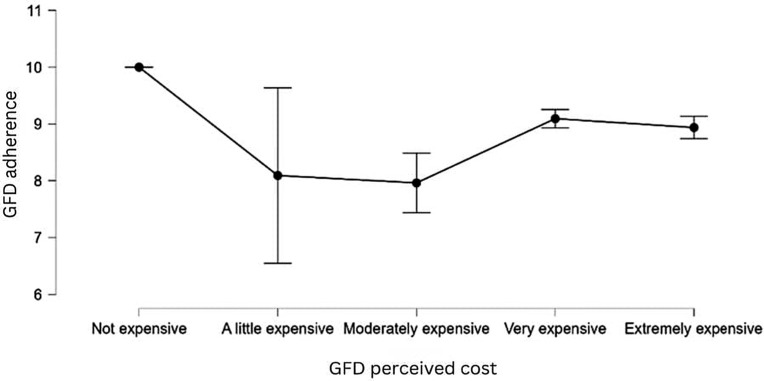
GFD adherence correlated with the perceived cost of GFD (GFD = gluten-free diet).

Our data shows that those who think that GF alternatives are moderately expensive are significantly less likely to adhere to the GFD compared to those who think that the GF alternatives are not expensive [*t* = −2.168, *p* = 0.030] (see Table 13 and Figure 5). There may be several factors contributing to this, and further investigation is required to comprehend this phenomenon. We did not identify any support for the patient categories that consider GFD alternatives to be “very expensive” or “extremely expensive”. Therefore, **H12** received partial support. Interestingly, we found high variability in adherence among the participants who rated the GFD as being “a little expensive”, while the other groups were more homogenous. The results of this analysis indicate a possible unknown third factor that affects GFD adherence.

A pertinent observation regarding the perceived expense of GFD is that a notably higher percentage of CD patients, specifically 50.09% of the total CD population, consider GF alternatives to be “very expensive”, in contrast to NCGS patients, who report this at a rate of 37.32%. This result is statistically significant [χ^2^ (4) = 39.725, *p* < 0.001].

**Figure 6:**
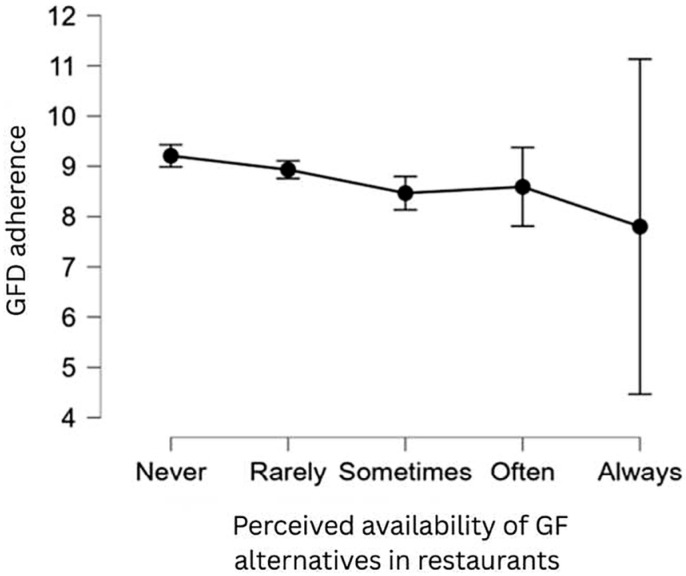
GFD adherence correlated with perceived GF alternatives in restaurant menus (GF = gluten-free; GFD = gluten-free diet).

Interestingly, our data show that a higher level of perceived availability of GFD alternatives in restaurant menus is correlated with lower GFD adherence (see Figure 6). Specifically, those who sometimes or always find GF alternatives in restaurant menus tend to have lower GFD adherence (see Table 13). However, these effects are considered small, according to Cohen (1988) [60] [*d* = 0.44 and *d* = 0.88 for “sometimes” and “always”, respectively]. We did not find a significant effect of the perceived availability of GF alternatives in stores on GFD adherence. These results do not support **H13**.

**Figure 7:**
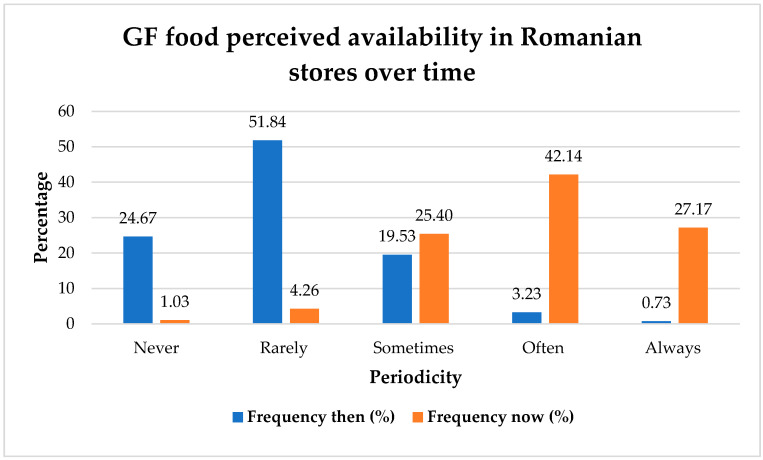
GF food perceived availability in Romanian stores over time: *then* at the time of diagnosis versus *now* at the time of questionnaire assessment (GF = gluten-free).

Since the study participants reported how often GF alternatives were found in stores, and as seen in Figure 7, comparative frequency data show a considerable improvement in the perceived availability of GF food in Romanian stores over time. The majority of respondents stated that they had limited access to GF items immediately after receiving the diagnosis, with 51.84% claiming infrequent availability and 24.67% saying they were never accessible. On the other hand, the data from the time of questionnaire assessment indicate a significant drop in both categories, with only 1.03% and 4.26% of respondents selecting “never” and “rarely,”, respectively. At the same time, the categories that indicate greater perceived availability have grown significantly: 42.14% of respondents now say that such items are “often” accessible, while 27.17% say they are “always” available, compared to just 3.23% and 0.73% in the past. Additionally, the “sometimes” group grew moderately, rising from 19.53% to 25.40%. These results point to a notable change in Romanian retail settings, favoring a more consistent and widespread access to GF foodstuffs, which may be a result of enhanced market response to special dietary requirements and possibly increased customer demand.

We found statistically significant associations between perceived food availability and the time since diagnosis, both for stores and restaurant menus. Specifically, for stores, we found a positive association between time since diagnosis and perceived food availability [χ^2^ (16) = 40.207, *p* < 0.001], in that more individuals than expected, sometimes, often, or always find GF alternatives as more time has passed since their diagnosis. With respect to restaurant menus, we also found a positive and statistically significant association between time since diagnosis and perceived GF food availability [χ^2^ (16) = 29.019, *p* = 0.024]. In this case, however, the increase in perceived availability is more prominent for those who were diagnosed more than 10 years ago, who, in a larger proportion than expected, responded that they sometimes find GF alternatives on restaurant menus. Thus, it seems that it is much harder for these patients to find GF foods in restaurant menus than it is to find them in stores.

Hypothesis **H14** was not supported by our data either, as we did not find any statistically significant differences in GFD adherence between the patients who learned about the GFD from a specialist (medical doctor/nutritionist-dietician) and those who learned about the GFD from other sources (self-study or other people with the same diagnosis). However, we identified statistically significant differences concerning the source of information (Table 14) between the CD and the NCGS patients [χ^2^ (3) = 15.657, *p* <0.001]: Significantly more CD patients (22.82%) than NCGS patients (12.68%) take their information from a medical doctor. Moreover, significantly fewer CD patients (48.98%) than NCGS patients (63.38%) find their information on their own. Finally, significantly more CD patients (22.63%) than NCGS patients (14.79%) learn about their condition from others with the same diagnosis.

The participants also gave information about the perceived difficulty of diet therapy. From those receiving education mainly from a specialist medical doctor/nutritionist-dietician (27.02%), 100% considered the GFD to be difficult or acceptable right after receiving the diagnosis. Moreover, 17.93% perceived their diet at the time of survey completion to be easy. Those who initially considered it difficult reduced from 84.24% to 19.56% by the time of assessment. From those receiving information about the GFD mainly from other people with the same diagnosis, or they relied on self-learning (72.98%), after diagnosis, 98.39% perceived the GFD to be difficult or acceptable (while 1.61% found it easy), but at the time of survey completion, 12.88% perceived it to be easy. The share of respondents who initially perceived it as difficult decreased from 81.89% to 20.72%.

Interestingly, 18.48% of those who received specialized education stated that there were situations where they ate gluten voluntarily, compared to the other group, where 17.10% agreed with this statement, making the second group less prone to making unsuitable dietary choices, although the difference between the groups is small.

**Figure 8:**
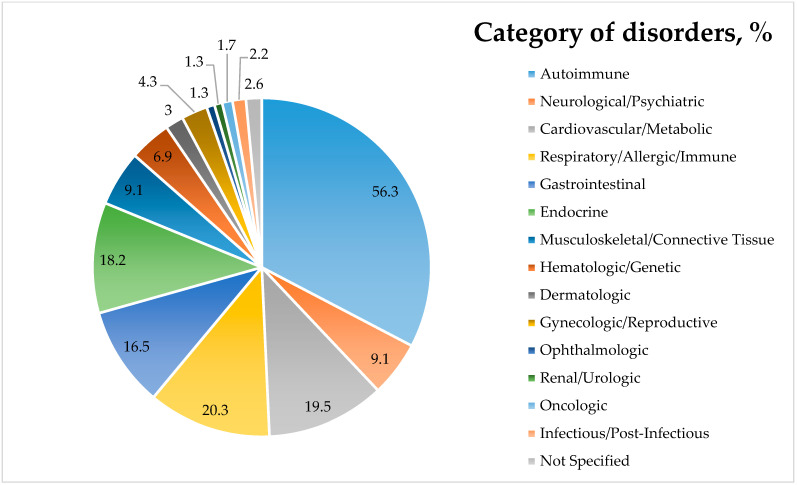
Reported chronic disorders by GRD patients.

In our study, 231 participants reported chronic associated conditions (excluding LI), with 168 diagnosed with CD and 63 with NCGS (Figure 8). Out of the 231 participants, six did not specify the particular chronic condition they have. As illustrated in Supplementary Table S1, between individuals with GRDs, a category of systemic conditions, particularly those stemming from both autoimmune and endocrine origins, is grouped together. Autoimmune disorders accounted for 56.3% of all comorbidities, indicating that they constitute the majority. Among these, autoimmune thyroiditis was most prevalent, affecting 32% of participants reporting comorbidities (18.6% autoimmune thyroiditis not otherwise specified, 12.1% Hashimoto’s, and 1.3% Basedow–Graves’). Type 1 diabetes was also significantly represented (5.6%), alongside conditions such as Sjögren’s syndrome (3.0%), psoriasis (2.2%), rheumatoid arthritis (2.2%), and Crohn’s disease (1.3%). These data corroborate theories about similar genetic and immunologic pathways and are consistent with previous research showing the significant co-occurrence of autoimmune disorders in people with GRDs, especially in CD.

The second most represented category was respiratory and allergic disorders, and they were reported in 20.3% of cases, with asthma (9.6%) and allergies (4.2%) being the most common. Cardiovascular and metabolic disorders collectively represented 19.5% of reported comorbidities, with arterial hypertension being particularly common (14.7%). Endocrine disorders were also prominently reported (18.2%), particularly hypothyroidism (7.4%), diabetes mellitus (3.9%), and prediabetes (2.6%). By totaling all thyroid dysfunctions, 42.4% of participants reporting comorbidities had such conditions. These data reinforce the well-established association between GRDs and endocrine dysfunction, with thyroid disorders being especially frequent.

Gastrointestinal (GI) conditions represented 16.5% of total comorbidities. Gastritis and duodenitis (4.3%), IBS (2.6%), gastroesophageal reflux disease (GERD, 2.2%), pancreatic insufficiency (1.7%), and hepatic disorders (1.3%) were most prevalent, suggesting persistent or secondary GI disturbances beyond GRDs. Interestingly, IBS was reported only by the NCGS group (9.5% of NCGS with reported comorbidities). These findings also emphasize the necessity of a thorough differential diagnosis in GRD patients presenting with persistent gastrointestinal signs and symptoms.

Neurological and psychiatric conditions were present in 9.1% of comorbidity cases, with neuropathy (1.7%), migraines (1.3%), and epilepsy (1.3%) among the most commonly reported. Other diagnoses included chronic fatigue, brain fog, anxiety, ADHD, autism, and others, findings that are consistent with accumulating evidence of extraintestinal neurological involvement in both CD and NCGS. Musculoskeletal and connective tissue disorders were reported in 9.1% of comorbidity cases, including osteoporosis (3.0%), cervical/lumbar disc herniation (1.3%), osteoarthritis (0.9%), and Strümpell–Lorrain disease (0.9%).

Hematologic/genetic (6.9%), gynecologic/reproductive (4.3%), dermatologic (3%), ophthalmologic (3%), renal/urologic (3%), infectious/post-infectious (2.2%), and oncologic conditions (1.7%) were reported at lower but clinically meaningful frequencies. Within these categories, endometriosis (3.5%) stands out as being more frequent, followed by anemia (2.2%), hepatitis (1.7%), alopecia (0.9%), and atopic dermatitis (0.9%).

For NCGS patients reporting chronic conditions, autoimmune thyroid diseases (30.2%) and high blood pressure (HBP, 19.0%) are the most common. For CD, the most common are also autoimmune thyroid diseases (32.7%) and HBP (13.1%).

Among the chronic conditions reported by SRGS study participants, 64.5% indicated having an autoimmune disease. Thyroid disorders were reported by 58.1% of respondents, while 25.8% reported having a gastrointestinal condition. Additionally, 19.4% reported high blood pressure, and 9.7% reported a diagnosis of endometriosis. These findings emphasize again that there might be a strong overlap between gluten sensitivity (even if it is self-reported) and other autoimmune or chronic conditions, highlighting the need for comprehensive clinical evaluation and a multidisciplinary approach to patient care.

Out of all participants included in this study, 19.2% reported having LI along with their GRD diagnosis (14.1% of those with CD, and 38.7% of those with NCGS), being a very common occurrence, especially for NCGS patients. The male-to-female ratio is 1:7.73 across diagnoses, and when distributed by diagnosis, it is 1:9.85 for CD and 1:5.87 for NCGS, making LI a more common occurrence for women with GRDs.

Overall, according to our study, people with GRDs have a significant burden of chronic comorbidities (33.9% of all responders to the questionnaire having at least 1 associated condition, different from LI). The systemic nature of GRDs and the significance of multidisciplinary screening, along with management strategies, are underscored by their frequent occurrence and diversity of associated conditions. The idea that GRDs are a component of a larger range of immune-mediated systemic illnesses rather than distinct gastrointestinal disorders is supported by this pattern of comorbidity.

In addition to the quantitative trends found, participants provided insightful firsthand accounts that reflected the practical, social, and emotional facets of following a GF lifestyle. It is anticipated that these stories, which will be thoroughly examined in a future, parallel qualitative research, will add important context to the current findings and shed further light on the lived experience of people with GRDs.

## 4. Discussion

When interpreting our findings in relation to the existing literature, it is important to acknowledge that studies on GRDs vary considerably in methodology, particularly with respect to diagnostic confirmation, sampling strategies, and data collection methods. Some of the most frequently cited studies rely on clinically verified diagnoses and structured clinical assessments, whereas our study is based on an anonymous, self-reported online survey. These methodological differences do not diminish the value of our findings but necessitate a cautious approach when comparing adherence rates, comorbidity profiles, or symptom patterns across studies. Accordingly, all comparisons in the Discussion are framed to highlight general trends and points of convergence or divergence rather than to imply direct methodological equivalence.

It has been reported that the general adherence to the GFD varies greatly from less than 50% up to 90% [61]. Unfortunately, there are still significant barriers preventing people from following a GFD accordingly. Some of the main issues that GRD patients face include restricted product accessibility, nutritional and dietary knowledge, high product costs, inadequate labeling or packaging, cross-contamination, therapy burden, inadequate awareness about GRDs and the GFD, psychological aspects, and more [62,63].

This research presents the inaugural thorough assessment of adherence to a GFD within a Romanian population suffering from GRDs, which includes both CD and NCGS. Our findings reveal that adherence is influenced by a combination of medical, psychosocial, and economic factors, with patients diagnosed with CD showing significantly greater adherence compared to those with NCGS. This observation is consistent with earlier studies that suggest the autoimmune characteristics of CD, the well-established mucosal damage caused by even minimal gluten intake, and the typically physician-directed diagnostic approach foster a stronger impetus for strict dietary adherence [3,4].

The GFD involves avoiding GC cereals: wheat, rye, and barley. Corn, millet, quinoa, buckwheat, and rice are all acceptable grains [64]. While oats are a GF option, they are often contaminated with wheat [65], and only the ones that are certified GF can be safely consumed. For foodstuffs to be classified as “gluten-free”, they have to contain less than 20 parts per million (ppm) of gluten [64]. It is acknowledged that ensuring an adequate daily nutrient intake for individuals following a GFD necessitates meticulous consideration of the nutritional attributes of the final product, particularly when eliminating GC ingredients from the formulation. Naturally, dairy, legumes, nuts, fruits, vegetables, meat, fish, and beans are all considered GF foods. The potential for gluten contamination arises when GF foods are exposed to GC products during the processes of manufacturing or preservation. Cross-contamination is a serious issue, and it has to be avoided for GRD patients, especially for those with CD [20].

Despite having distinct pathogenetic processes that contribute to their development and very similar clinical symptoms, CD and NCGS benefit from the same type of diet therapy [34]. Patients with CD must follow a very strict GFD since even trace levels of gluten can cause the intestinal mucosa to be negatively affected [38]. Interestingly, compared to patients with CD, individuals with NCGS have a lower adherence to the GFD [35], and our study findings underscore this phenomenon. Furthermore, GFD is still the best treatment for improving the clinical symptoms of people with NCGS, although this diet may not be as rigorous as that for patients with CD, and combining it with a low-FODMAPs diet is suggested [32,38]. Neither diet is recommended for healthy individuals, as they may bring nutritional and intestinal microbiota imbalances, as well as malnutrition and adverse long-term consequences [32]. Adhering to a GFD leads to the remission of signs and symptoms in both instances; however, these manifestations reappear upon the reintroduction of gluten [24]. Therefore, good adherence to diet therapy is essential for improving the quality of life (QoL) of those with GRDs and avoiding complications.

A notable concern is the considerable number of self-diagnosed individuals, particularly within the NCGS group. Self-diagnosis was found to be independently linked to markedly lower adherence to the GFD, supporting previous findings that this practice heightens the risk of misclassification, nutritional deficiencies, and insufficient dietary restrictions [66]. This highlights the critical need for formal diagnostic procedures prior to the commencement of lifelong restrictive dietary interventions.

Self-reported gluten sensitivity (SRGS) is increasingly problematic, as it compels patients to adhere to a GFD, which is highly restrictive. This situation not only heightens the risk of misdiagnosis but also leads to potential complications and adverse effects for the patients involved [67]. It is more common in NCGS patients, where most of them rely on self-diagnosis [68]. Our results support this idea. Approximately 11% of questionnaire responders reported self-diagnosing themselves. Among them, 80% are NCGS patients and 20% are CD patients, pointing to this practice as being predominant in the NCGS group. Such practice should be strongly discouraged. Moreover, this difference may be explained by the availability of clear and validated diagnostic criteria for CD (including serological and histological testing), while NCGS lacks specific biomarkers and a gold standard for diagnosis, with the diagnosis usually based on the exclusion of CD and WA, combined with clinical improvement on a GFD and relapse on re-introduction of gluten. The milder and often non-specific nature of NCGS symptoms (bloating, fatigue, headache) leads patients to more often resort to self-treatment based on Internet sources and personal experience rather than seeking medical advice. This carries the risk of missing CD or other gastrointestinal diseases and may complicate future diagnosis if a GFD is initiated before the necessary tests have been performed [35].

SRGS is substantially linked to IBS in contrast to the general population [66]. In one study, for instance, 37% of people with SRGS matched the Rome III criteria for IBS, but just 9% of those in a control group did the same [69]. According to different research, 44% of those who self-reported having non-celiac wheat sensitivity (NCWS, a designation that is sometimes used synonymously with NCGS) also had symptoms of IBS [70]. In a controlled trial, only one-third of SRGS participants were confirmed to have NCGS, while the others were self-misdiagnosed [71]. In our study, we found that the participants who self-diagnosed and also reported having IBS as an associated condition are all part of the NCGS category. Furthermore, the fact that IBS only occurs in NCGS (regardless of the diagnosis criteria) highlights the question of whether these conditions are symptoms of a common intestinal hypersensitivity spectrum or separate pathophysiological processes.

Self-diagnosis is highly inadvisable and, regrettably, it occurs with notable frequency. Research indicates that approximately 13% of the British population engages in this practice [67], while in the Netherlands, the prevalence is lower at around 6% [20]. In Argentina, the rate is estimated to be nearly 8% [65]. This approach to self-diagnosis, linked to the adherence to a GFD, is frequently adopted by individuals who experience relief from their symptoms upon the removal of GC foods from their diet [39]. Cabrera-Chávez et al. conducted a survey study reporting that weight loss and the belief that a GFD is healthier are actually the primary drivers of GFD adherence, observing that at least 50% of the GF participants in this research are following their diet without consulting a medical doctor or dietician [66]. However, research based on CD patients’ experiences indicates that following a GFD, their weight is more inclined to rise [35], and it is untrue to say that eliminating gluten improves general health or that it is better than the typical standard diet [32,35]. Our research focused on the Romanian population, which exhibited an 11% rate of self-diagnosis, indicating that weight fluctuations after initiating a GFD are significantly associated with adherence to the GFD. Specifically, individuals who reported weight gain demonstrated a higher level of adherence. The weight gain can be due to a multitude of factors, including an imbalanced nutritional profile with many nutrient deficiencies that might result from adhering to a GFD. This happens mostly because important sources of fiber, vitamins, and minerals are eliminated when wheat, rye, and barley are excluded. Many GF products are composed of refined flour or starches that are generally not enriched, potentially resulting in lower levels of essential micronutrients such as calcium, iron, zinc, magnesium, folate, and vitamins D and B. Furthermore, GF foodstuffs often contain elevated amounts of fat, salt, sugar, and simple carbohydrates, contributing to an overall nutritional imbalance. Consequently, these dietary inadequacies may increase the risk of metabolic disorders [20]. To guarantee that those following a GFD fulfill their daily nutritional intake requirements and have balanced eating habits, dietary advice and, in certain situations, food fortification or supplementation are advised due to these difficulties [20,29,65].

According to our results, the adherence with the GFD is strongly predicted by the reported diagnosis (CD versus NCGS) as well as the diagnosis approach (specialized versus self-diagnosis). Those with CD had much higher adherence than the NCGS group. Given that patients with confirmed CD are usually given organized dietary advice and are better aware of the long-term health concerns of gluten consumption, this trend probably reflects variations in how the disease is perceived, how symptoms may be predicted, and medical monitoring. On the other hand, patients with NCGS can have erratic symptoms and less clinical monitoring, which would lead to less reliable adherence. Similarly, those who self-diagnosed reported less adherence than those who had an official diagnosis. These results emphasize the value of expert assessment, instruction, and continuing assistance for those following a GFD. Without medical validation, there may be less incentive to follow through rigorously, especially if symptom response is unclear. The lack of substantial correlation between adherence and other individual characteristics, such as time since diagnosis, comorbidity status, and family history of GRDs, suggests that qualified supervision and diagnostic precision may be more important than chronological or interpersonal variables. Collectively, these findings demonstrate how important medical care and diagnostic assurance are to maintaining long-term GFD adherence across the range of GRDs.

All in all, self-diagnosis is consistently associated with lower adherence to the gluten-free diet because individuals without a formal medical diagnosis typically lack access to structured medical guidance, dietetic counseling, and follow-up—factors known to improve long-term adherence. Without diagnostic confirmation, symptoms may also be perceived as less severe or more ambiguous, reducing motivation to maintain a strict diet. In addition, self-diagnosed individuals often have less knowledge of hidden gluten sources, fewer opportunities for clinician-reinforced education, and may experience greater uncertainty about the necessity or strictness of the diet. These factors collectively contribute to lower adherence in self-reported GRD cases, a pattern that has also been noted in previous research [35,41,66,68,70,71,72,73,74].

In our study, label-reading practices emerged as one of the most significant predictors of adherence, regardless of the span of time after diagnosis. Interestingly, the CD group practices label reading more often and is more adherent to the GFD compared with the NCGS group. However, over a quarter of participants reported experiencing symptoms after consuming products labeled as GF. This indicates potential shortcomings in the accuracy of product labeling and the control of cross-contamination, issues that have been well documented in other national settings [75]. There is a pressing need for more stringent certification oversight and alignment with European GF standards to ensure the integrity of dietary practices.

In their recent study, Jeanes et al. highlighted an issue regarding the difference in the confidence levels of people with CD who are following a GFD when they buy food at supermarkets versus online. Although most people (58%) feel comfortable reading and understanding food labels in stores, a much smaller portion of patients (38%) have the same level of confidence when they purchase online [76]. This discrepancy suggests potential challenges regarding the accessibility or clarity of food labeling, which could adversely affect the safety and dietary adherence of these patients. The sociopsychological load of a GFD is further increased by difficulties in recognizing real GF items, understanding food labels, and navigating dietary restrictions when traveling or attending social gatherings [67]. A UK-wide cross-sectional study drew attention to the important role that medical professionals play in facilitating patient self-management [76]. Educational programs, awareness campaigns, and counseling are crucial to assist in promoting diet adherence. According to Cheng and Handu, thorough nutrition evaluation, instruction, and continuing counseling are essential for giving patients the knowledge and confidence they need to follow their diet correctly [65]. Therefore, in addition to legislative initiatives to improve the accuracy and comprehensibility of GF labeling, label literacy should be incorporated into patient education initiatives. Adherence rates and general health among people with GRDs can be significantly increased by enhancing systemic labeling requirements and patient education.

There was no evidence in our findings to support the hypothesis that professional nutritional education would increase dietary adherence. Patients who received expert education and those who acquired knowledge on their own showed comparable levels of adherence, which may indicate differences in the caliber or accessibility of counseling. Nonetheless, prior studies emphasize that expert advice is still necessary for long-term adherence and self-assurance in diet management. Several testimonies emphasized the benefit of expert advice, even if quantitative results did not reveal any appreciable variations in adherence depending on the source of nutritional information. One respondent supplied an explanation: “I found out from Facebook groups about a doctor who referred me to a gastroenterologist in Bucharest … I believe it is important for doctors to have knowledge of CD, for patients to be referred to nutritionists who have knowledge about these conditions, and also for them to be information about the existence of ARIG.”

The Romanian population affected by GRDs can receive support from the Romanian Association for Gluten Intolerance (Romanian: Asociația Română pentru Intoleranță la Gluten, ARIG). ARIG is the only national organization in the country dedicated to individuals with such disorders. It has received accreditation from the Association of European Celiac Societies (AOECS) and has the endorsement of the Regional Center for Celiac Disease Management in Bucharest [77,78,79,80]. ARIG plays a crucial role in patient advocacy and education. At a national level, improving collaborations among such organizations, medical professionals, and legislators may improve the patient’s experience and dietary adherence.

Furthermore, our findings presented in Table 14 suggest that individuals diagnosed with CD and NCGS predominantly depend on self-obtained information when seeking to understand the GFD they are required to adhere to following their diagnosis. Remarkably, 48.98% of CD patients and an even higher 63.38% of NCGS patients said they learned about GFD on their own, suggesting that they did not access expert dietary guidance very often. Just 5.57% and 9.15% of CD and NCGS patients, respectively, reported receiving the information from a specialized nutritionist–dietitian, while only 22.82% and 12.68% of patients, respectively, named doctors as their primary source. These results point to possible deficiencies in the availability or accessibility of professional nutritional assistance, especially for those with NCGS who could encounter less organized diagnostic and therapeutic processes. Additionally, 22.63% of CD patients and 14.79% of NCGS patients rely on information from other patients who have the same condition, demonstrating the continued importance of peer networks.

Among CD patients, peer support is more prevalent, which probably reflects more robust advocacy networks. The excessive dependence on unofficial sources, such as peer recommendations and self-sourced information, raises questions regarding the reliability and security of the data influencing GFD adherence and points to serious gaps in access to expert dietary counseling. Therefore, the data also highlight the necessity of better educating healthcare providers on evidence-based dietary recommendations in order to guarantee reliable and efficient GFD adherence.

We can therefore observe that nutritional counseling was not utilized to its full potential, with the majority of participants engaging in self-directed learning. Moreover, the responders to our questionnaire were also asked about the perceived difficulty of diet therapy. The results lead us to the conclusion that specialized diet therapy instruction is crucial in influencing patients’ attitudes to make converting to a GFD easier. Professional nutritional counseling improved long-term perceptions of dietary management, even though almost all patients first found it difficult. Nevertheless, even in the absence of professional guidance, patients gradually adjusted, and the proportion of those who found the diet difficult dropped considerably. However, specialist nutritional instruction seems to help patients better adjust and may improve overall adherence and the results of dietary management. A multidisciplinary team—including a clinical dietitian/nutritionist, gastroenterologist, and psychologist—can conduct such training. A clinical pharmacist also plays an essential role in addressing food–medicine interactions and ensuring that all patient needs are met [81,82,83,84].

Notably, the percentages of participants who intentionally consumed gluten were very similar in the two groups (18.48% for those receiving specialist instruction vs. 17.10% for those relying on self-education). Therefore, it may be vital to investigate additional motivational or psychosocial factors influencing these patients’ voluntary gluten intake, as specialized dietary education alone may not be enough to prevent intentional dietary deviations. According to the survey study of Silvester et al., whose findings are consistent with ours, CD patients are more likely to adhere rigorously to the GFD, without eating gluten intentionally [75]. The study in question also revealed a noteworthy finding: individuals diagnosed with CD tend to obtain information primarily from medical doctors, while also seeking out a variety of supplementary resources, such as organizations dedicated to CD and various relevant publications. In contrast, the non-CD group is more inclined to gather information from practitioners of alternative healthcare (as well as from other individuals on a GFD) and less likely to have learned of a GFD from healthcare professionals and celiac assistance groups or associations. It was also observed that there is a tendency to avoid eating outside of their home (e.g., restaurants, other people’s homes) in CD patients [75]. Additionally, our study shows that people who are less socially active also follow the GFD more closely, which implies that social exposure is a major obstacle to rigorous adherence. Dietary lapses are more likely to occur in patients who regularly attend social gatherings without GF choices. One participant shared: “Since diagnosis…I haven’t gone out to restaurants, to weddings, birthdays, or with friends, because I can’t go with the food casserole from home, and I wouldn’t feel good at all. Everything comes down to eating at home, unfortunately. The level of frustration is really high.” This underlines the crucial role of public awareness campaigns and social inclusion tactics in lowering stigma and promoting the mental health of patients. The psychological cost of therapy adherence is further highlighted by this trend, which emphasizes the need to create environmental and social assistance—such as increased GF options and social education—to create a balance between social inclusion and adherence. Regarding coping mechanisms in social situations, an intriguing behavioral variation between the diagnostic groups has been identified. Compared to people with NCGS (77.46%), a significantly higher percentage of individuals with CD (91.28%) reported bringing their own food to social gatherings. This conduct probably reflects a higher awareness of the gravity of the disease and the need for tight adherence, as well as increased alertness and knowledge of cross-contamination dangers among CD patients.

Food is vital for social connections and general well-being in addition to providing necessary sustenance. Although avoiding social situations may lower the risk of accidental gluten exposure, it can also intensify feelings of isolation, frustration, a sense of being misunderstood, and diminish QoL, a concern often highlighted in literature discussing the psychosocial challenges of therapeutic diets [67,85]. According to Silvester et al., individuals adhering to this diet experience the most significant adverse effects on their leisure and social engagements when contrasted with non-CD patients who are following GFD, the majority of whom are individuals with NCGS and possess a limited understanding of the implications of this dietary approach. Furthermore, the GFD can lead to feelings of anxiety, discomfort, or embarrassment during social dining situations. Social pressure significantly influences the levels of adherence to therapeutic regimens, particularly among patients with CD, who may feel compelled to consume GC foods due to the expectations of their social environment. Furthermore, stringent dietary guidelines can stifle spontaneity and may be perceived as a rejection of hospitality or an imposition of limitations [75]. Consistent with this, over 65% of our participants indicated experiencing perceived discrimination related to their condition, which was associated with decreased involvement in social activities. Notably, a higher number of individuals with CD refrained from participating in social situations compared to those with NCGS. This observation suggests a deeper awareness of the condition as well as an increased apprehension regarding inadvertent gluten exposure.

The perceived significance of the GFD—at both the time of diagnosis and when the survey was completed—showed a positive correlation with adherence. In line with earlier research showing the impact of motivation, disease awareness, and perceived necessity on long-term dietary behavior [75,86], people who thought the GFD was “very important” showed noticeably higher adherence than those who thought it was of less importance. The data also showed that the perceived importance of GFD increases over time, accompanied by improved adherence. This likely reflects the cumulative effects of symptom relief, experience, and continued education or peer support. GFD adherence was not independently predicted by perceptions of dietary difficulty, discrimination, or acceptance of the diagnosis; however, these factors probably interact with wider psychological processes that influence disease adjustment. Patients’ long-term adaptability and increasing self-efficacy may be the reason for the lack of notable impacts. The challenging social costs of rigorous adherence are highlighted by the found correlations between social disengagement and felt discrimination. This highlights again the need for patient-centered therapies that address the social and emotional aspects of living GF.

Although the perception of the GFD difficulty did not directly predict adherence, our supplementary analysis revealed meaningful patterns. Overall, perception tends to enhance considerably as time progresses, transitioning from a state of being “difficult” to predominantly “acceptable” or even “easy” for numerous individuals. Naturally, adaptation occurs over time. Notwithstanding this progress, CD patients still view the diet as being much more difficult than NCGS patients, underscoring lingering difficulties and possible disparities in the two groups’ experiences and perceptions, pointing to the idea that a very strictly followed diet and high adherence (aspects more prominent in the CD group) make the experience more difficult. This likely reflects the stricter requirements and higher cost of non-adherence in CD, where even minimal amounts of gluten can cause intestinal damage and long-term complications [87,88]. Additionally, it emphasizes the need for focused assistance and guidance to improve adherence and QoL for CD patients, particularly in the early phases of dietary transformation.

The notable rise in the popularity of GF products and the substantial growth of the GF industry are indeed impressive; however, the general population remains largely uninformed about gluten and GRDs. Furthermore, a limited proportion of SRGS individuals adhere to a GFD as recommended, with issues related to cost and accessibility posing significant challenges [39]. According to another British study, although GF food is readily available in reputable, everyday shops and online, the cost is still much higher. Interestingly, no GF goods are carried by low-cost stores, which are typically visited by patients from lower socioeconomic groups [89]. Furthermore, GF goods can be, on average, 242% more costly than conventional goods [67]. In our findings, it is clear that economic obstacles continue to pose a significant challenge. The results provide partial support for the hypothesis that perceiving GF products as expensive is associated with lower dietary adherence. Participants who viewed GF products as moderately costly demonstrated lower adherence levels than those who considered them affordable, while perceptions of extreme expense did not show the same trend. This may suggest differences in coping or purchasing behaviors. Interestingly, over 50% of the CD participants rated GF products as very expensive, and this aligns with prior research highlighting the economic burden of strict gluten avoidance and its impact on dietary adherence and QoL. One potential explanation for the perceived elevated cost of the GFD may stem from the strict adherence to purchasing only certified foods or appropriate alternatives for the GFD. This practice could result in a significantly greater financial impact, as the GFD tends to be considerably more costly than a GC diet.

Our study findings did not show a significant effect of the perceived availability of GF alternatives in stores on GFD adherence. On the other hand, they show that higher perceived availability of GF alternatives in the restaurant menus correlates with lower adherence to GFD, which is the opposite of what we initially hypothesized. A possible factor could be regarding the social situations and peer pressure: people may dine out more frequently when they believe that GF options are readily accessible, which exposes them to more circumstances where following stringent GF guidelines might be difficult (heightened risk of cross-contamination). It is also possible that participants misreported their adherence, availability, or both. The relationship could also be skewed by a gap between the perceived and actual availability of GF items. Various factors may contribute to this issue, and further investigation is required to comprehend this phenomenon.

We also observed that, especially in retail establishments, the perceived availability of GF goods in Romania has significantly improved over time. Following diagnosis, the majority of participants originally reported having little to no access to them; however, at the time of survey completion, the responses indicate a significant change, with over 40% now stating that these products are “often” accessible and over a quarter saying that they are “always” available. This pattern demonstrates that the Romanian food market has responded to customer demands and dietary variations over time, which is probably a reflection of larger worldwide advancements in the availability and labeling of GF products. Despite this improvement, participants continued to believe that there were fewer GF alternatives available in restaurants than in supermarkets, with statistically significant correlations indicating that even those with a long-term diagnosis frequently had trouble finding appropriate meals while dining out. Although GF items are now more available in retail settings, the hospitality industry still needs to be upgraded, especially through employee education, clearer menu labels, and cross-contamination avoidance strategies.

One participant in the study stated, “I always want to eat GC products, they seem better to me and I’m tired of the few products that are available in some stores, which are also very expensive.” This demonstrates the intersection of economic and sensory discontent, highlighting the reality that expensive and limited access can eventually weaken motivation in addition to lowering dietary adherence. Overall, the limited availability and increased costs of GF products significantly affect adherence among underprivileged populations. To ensure that diet therapy is accessible to all, it is essential to reduce prices. Furthermore, it is crucial to evaluate policies regarding the delivery of therapy across different socioeconomic groups and to implement strategies aimed at reducing disparities in access and outcomes [89,90]. Collectively, our findings align with global trends in GFD adherence while also highlighting distinct dynamics specific to Romania, such as a swift enhancement in the availability of retail GF products and ongoing shortcomings in professional dietary guidance.

It is important to emphasize that CD is a disease that can occur at any age. Women show a higher incidence of CD and other autoimmune reactions to gluten, and this is well documented in the scientific literature, with the gender difference being seen in both children and adults. Interestingly, individuals with CD who exhibit greater intestinal damage also demonstrate this gender disparity. The reasons are multifactorial and include immunological, hormonal, and genetic factors. According to epidemiological data, most population studies show that in adults, the female–male ratio is about 2–3:1. In a meta-analysis by Singh et al., the incidence of diagnosed cases was 0.6% in men versus 1.1% in women. Possible mechanisms are both hormonal influences and genetic factors. People with CD have HLA-DQ2 and/or HLA-DQ8 alleles. Although the distribution of these alleles is similar in both sexes, women have a higher expression of additional immunoregulatory genes on the X chromosome that are associated with autoimmunity. From an immunological point of view, women have a more active T-cell response to antigens, including gluten peptides. Increased production of autoantibodies is more common in women even before the onset of clinical symptoms. Last but not least, the fact that women are more likely to seek medical help and undergo tests when they have symptoms increases the number of diagnosed cases [1,4,91,92,93,94,95,96,97,98]. The strong heredity of CD is highlighted by Salazar et al., being suggested that first-degree relatives of CD patients are far more likely to develop CD (having a significantly higher chance than second-degree relatives), with a 10–15% prevalence [99], as do those with type 1 diabetes mellitus, other autoimmune disorders, Down syndrome, and several other related conditions [1,3]. Moreover, Dochat et al. conducted a survey study on self-reported, biopsy-confirmed CD U.S. adults, showing that 27.8% had LI as an associated condition [100]. Research involving relatives of individuals with CD and the identification of numerous associated disorders provides valuable insights in the search for oligosymptomatic cases, particularly when patients exhibit atypical symptoms. Adequate adherence to a GFD, particularly if started early, may reduce the progression of additional problems and/or related disorders [30].

It is believed that approximately 12% to 25% of NCGS patients have first- or second-degree relatives with CD, and it affects more women than men [38]. An Italian study that analyzed 486 individuals with suspected NCGS revealed a 5.4:1 female-to-male ratio, and it was also observed that 14% of them had an associated autoimmune disease (mainly autoimmune thyroiditis). Interestingly, other disorders were associated with NCGS as well, with 47% displaying IBS, 35% with food intolerance (predominating LI), and IgE-mediated allergies in 22% of cases studied. The same study reported that 18% of NCGS patients have first- or second-degree relatives with CD, which is consistent with other studies [68]. Furthermore, Molina-Infante et al. indicate that as many as 20% of initial cases of NCGS may subsequently be reclassified as CD following additional testing and evaluation. This finding underscores the critical necessity of ruling out CD prior to establishing a diagnosis of NCGS in patients [42].

In our study, the majority of responders stated having CD (almost 80%), and those with NCGS represent a significantly smaller group. Regarding gender distribution, the male-to-female ratio is 1:5.36 across diagnoses. For CD, M:F = 1:5.34, which underlines a greater female dominance compared with other studies, and M:F = 1:5.45 for NCGS, which is consistent with previous findings. These findings are consistent with other Romanian findings, and according to the study of Balaban et al., the female predominance of the CD diagnosis can be seen in both adults (~71%) and children (~67%) [22].

Genetics plays a crucial role, and hereditary factors are obvious in CD, but NCGS does not seem to have a significant hereditary foundation [3]. We also analyzed the hereditary factor and observed that almost a quarter of the participants in this study reported having family members with the same diagnosis as themselves [$\chi^{2}\;=\;4.017,\;p=0.045$]. Interestingly, this seems to be a more common phenomenon among the NCGS group (28.9%) compared with those stating having CD (20.97%). According to another Romanian study, when looking at the family history, it was observed that 10.53% of the participants had a first-degree relative diagnosed with CD [22]. While CD has a well-established genetic component and NCGS does not have a clear genetic process or specific marker, there is also no concrete proof that NCGS and CD are inherited similarly [101]. A significant factor may be the interplay between genetic predisposition and common environmental and dietary practices. Our research highlights the well-known risk associated with GRDs and may suggest a greater hereditary influence within the Romanian population. This finding underscores the necessity for increased testing, the implementation of national screening initiatives, and further investigation to enhance our understanding of this phenomenon.

As previously mentioned, GRDs are frequently associated with other pathologies. The comorbidity profile identified in this cohort (see Figure 8 and Supplementary Table S1)—characterized mainly by autoimmune thyroid disease, hypertension, allergic conditions, endocrine and gastrointestinal disorders—reinforces the notion of GRDs as systemic immune-mediated conditions rather than merely isolated gastrointestinal issues. Autoimmune conditions were reported in more than half of the cases, which raises a serious concern and burden for GRD patients, also emphasizing the need for a thorough diagnosis process and patient assessment. Hypothyroidism, diabetes mellitus, and hypertension were among the common endocrine and metabolic conditions, indicating that gluten-related autoimmunity could interact with more extensive metabolic and cardiovascular risk pathways. The significant presence of neurological and psychiatric comorbidities, including migraine, neuropathy, and cognitive symptoms, contributes to the growing body of data suggesting that GRDs may spread outside the gastrointestinal tract, possibly through gut-brain or neuroimmune pathways. Overall, these results demonstrate the necessity of managing GRDs using a multidisciplinary strategy that incorporates knowledge from the fields of gastroenterology, endocrinology, neurology, and nutrition. Additionally, they support the idea that GRDs are not distinct enteropathies but rather one aspect of a larger network of immune-mediated systemic diseases.

The significant prevalence of LI, especially among NCGS patients, adds complexity to dietary management and underscores the necessity for integrated, multidisciplinary care strategies. LI is seen as a frequent occurrence among GRD patients, and our study emphasizes this idea. Moreover, our results suggest that LI is more likely in women with GRDs, and they raise the question of whether LI can be used as a possible sign helping the diagnosis process, since 19.2% of all responders reported having LI along with their GRD diagnosis. Another question that is being raised is whether practitioners should test the presence of GRDs for patients diagnosed with autoimmune thyroiditis (since this is the most common comorbidity, with 32% occurrence among patients who reported having a comorbidity). Extensive research is needed to develop a more detailed and precise diagnosis approach.

A thorough thematic analysis of all gathered experiences from the participants in this study will be covered in a future qualitative research, even if just a small number of testimonials were employed here to set the quantitative results in perspective.

### Limitations

It is important to acknowledge the limitations of this study. Self-reported data might have included recollection or social desirability bias, especially with regard to dietary adherence, and the cross-sectional design precludes a determination of causal correlations between variables. The online format might have favored the inclusion of people with higher digital literacy, and who were more familiar with GRD support groups and the GFD. Therefore, the study only covers the Romanian GRD community’s internet-connected members, not the overall population. Furthermore, diagnostic verification relied on self-declaration, which might have limited the comparability of the CD and NCGS groups. Lastly, these findings should be considered cautiously since they may not be entirely transferable to other nations as they represent the unique cultural, economic, and healthcare environment of Romania. Despite these limitations, the reliability of the observed patterns is strengthened by the large sample size and broad spectrum of medical, behavioral, and economic factors. These constraints affect the generalizability of the findings but do not compromise the internal consistency or statistical validity of the analyses conducted within the surveyed population.

## 5. Conclusions

In this online, questionnaire-based cross-sectional study of a Romanian cohort (*n* = 681) living with GRDs, our findings show that self-diagnosis, perceived cost, and social involvement obstacles have a negative impact on dietary adherence, while diagnostic certainty, time since diagnosis, and perceived importance of the GFD are significant variables that influence dietary adherence. These results highlight that following the GFD is a complex journey and how psychosocial variables play an essential part in it. Our study also emphasizes the necessity of organized, interdisciplinary approaches that incorporate psychosocial support, dietary counseling, and medical advice. To encourage long-term adherence, policy changes that enhance the accessibility, price, and labeling of GF foods are also crucial. The high prevalence of comorbid autoimmune, endocrine, cardiovascular, respiratory, and allergic disorders highlights the systemic nature of GRDs, emphasizing the necessity for thorough clinical assessments and multidisciplinary management approaches. Additionally, the elevated rates of LI, particularly in NCGS, necessitate dietary planning that accommodates multiple simultaneous restrictions.

Our analysis provides insightful information about managing GFDs in the real world. Future research should investigate longitudinal patterns of adherence, the relationship between psychosocial resilience and dietary adherence, and the efficacy of targeted interventions—especially those that integrate nutritional education with social and economic support—in promoting sustained long-term adherence across various GRD populations.

### Future Perspectives

To enhance dietary adherence and optimize patient outcomes, we suggest that public health, clinical strategies, and research concentrate on the following key priorities:

1. Strengthening diagnostic pathways to reduce self-diagnosis and guarantee the proper initiation of restrictive diets
2. Improving the accuracy of food labeling and oversight of certification to minimize unintentional gluten exposure.
3. Increasing access to specialized nutritional counseling as a fundamental aspect of GRD care and management.
4. Addressing economic obstacles through policy initiatives aimed at alleviating the financial burden associated with GF products.
5. Incorporating psychosocial support to alleviate feelings of isolation, discrimination, and impairments in QoL.
6. Examining and identifying feasible strategies to expand dietary and nutritional support resources.
7. Assessing long-term dietary adherence trends and the evolving influence of clinical, emotional, financial, and economic variables on persistent GFD adherence.
8. Investigating how improved referral procedures and standardized diagnostic processes may discourage self-diagnosis and increase GFD adherence.
9. Comparing Romanian data with findings from other nations in order to acquire a better understanding of how cultural and health-system variations affect the oversight of GRDs and GFD adherence.

## Figures and Tables

**Table 1:** Summary and classification of CD manifestations and related conditions [1,3,7,9,10,24,25,26,27,28,29,30].

CD Manifestations and Related Conditions	
**Gastrointestinal** **Manifestations**	Diarrhea, constipation, vomiting, bloating, abdominal distension, abdominal discomfort and pain, flatulence, nausea, loss of appetite, acid reflux, dyspepsia
	Intestinal inflammation, intestinal malabsorption and maldigestion, nutrient deficiencies, altered nutritional status
	Lactose intolerance (in ~50% of patients at diagnosis) and intolerance to other carbohydrates
	Vitamin, mineral, and other deficiencies (B vitamins, including folate; vitamin D; calcium; zinc; iron; ferritin) due to malabsorption
**Extraintestinal** **Manifestations &** **Related** **Conditions**	**Cardiovascular:** pericardial effusion, myocarditis, cardiomyopathy, autoimmune pericarditis
	**Musculoskeletal:** osteopenia, osteoporosis, osteomalacia, muscle wasting, myalgia, arthritis
	**Dermatological:** atopic dermatitis, eczema, chronic urticaria
	**Reproductive:** infertility, recurrent miscarriage, amenorrhea, delayed menarche, early menopause
	**Hepatological:** hepatitis, transaminitis
	**Neurological:** headaches, migraines, epilepsy, cognitive impairment (“brain fog”), dementia
	**Oral:** dental enamel defects, aphthous stomatitis
	**Psychiatric:** anxiety, depression, fatigue, dysthymia, behavioral disorders, autism spectrum disorders, ADHD, eating disorders
	**Autoimmune endocrine disorders:** thyroid diseases such as autoimmune thyroiditis, mainly Hashimoto’s, Graves’ disease; Addison’s disease; type 1 diabetes mellitus
	**Autoimmune dermatological diseases:** dermatitis herpetiformis, vitiligo, alopecia areata, dermatomyositis, psoriasis, alopecia areata
	**Autoimmune neurological diseases:** gluten ataxia, peripheral neuropathies
	**Autoimmune liver diseases:** autoimmune hepatitis, primary biliary cirrhosis, primary sclerosing cholangitis, Wilson’s disease, Budd-Chiari syndrome
	**Rheumatological and connective tissue diseases:** Sjögren’s syndrome, systemic lupus erythematosus, systemic sclerosis, rheumatoid arthritis, and idiopathic inflammatory myopathies
	**Other:** iron-deficiency anemia, food intolerances, Down syndrome, pancreatitis, weight loss, short stature in children

**Table 2:** Summary and classification of NCGS manifestations and related conditions [3,12,34,36,38,40,41,42,43,44].

NCGS Manifestations and Related Conditions	
**Gastrointestinal** **Manifestations**	Diarrhea, constipation, nausea, vomiting, bloating, abdominal distension, flatulence, acid reflux, dyspepsia, epigastric pain, abdominal discomfort and pain, pyrosis, aerophagia
	Intestinal inflammation, nutrient deficiencies, altered nutritional status
	Food intolerances (mainly lactose intolerance)
	Vitamin and mineral deficiencies (B vitamins, including folate; vitamin D; calcium; iron)
**Extraintestinal** **Manifestations &** **Related** **Conditions**	**Psychiatric:** anxiety, depression, behavioral or mood disorders, sleep disorder
	**Neurological:** headaches, gluten ataxia, cognitive impairment (“brain fog”), fatigue
	**Musculoskeletal:** myalgia, arthritis, fibromyalgia, limb numbness
	**Dermatological:** dermatitis herpetiformis, atopic dermatitis, eczema, rashes, psoriasis, urticaria
	**Reproductive:** menstrual disorders
	**Oral:** aphthous stomatitis
	**Other:** weight loss, autoimmune disorders (such as autoimmune thyroiditis), allergic rhinitis, food allergies and intolerances, IgE-mediated allergies, rhinitis, asthma, anemia, IBS

**Table 3:** Study hypotheses.

Hypotheses	Medical and Individual Factors
H1	Patients who have relatives with the same condition as themselves show higher adherence to the GFD.
H2	Patients with CD exhibit greater adherence to the GFD compared to those with NCGS.
H3	Time since diagnosis is positively correlated with dietary adherence (patients diagnosed longer ago adhere more strictly to the GFD).
H4	Patients with multiple chronic conditions demonstrate higher adherence to the GFD than those without comorbidities.
H5	An official medical diagnosis increases the likelihood of dietary adherence compared to self-diagnosis.
H6	Patients who frequently read/check food labels show greater adherence to the GFD. There is an interaction effect between food label reading and time since diagnosis on dietary adherence.
	**Psychosocial Factors**
H7	Personal acceptance of the diagnosis positively influences adherence to the GFD.
H8	Patients who perceive the GFD as difficult are more likely to be non-adherent.
H9	Patients who perceive the GFD as more important are more likely to be adherent to it.
H10	Frequent attendance at social events without GF options is associated with lower dietary adherence. Those who are more adherent to the GFD tend to isolate themselves more.
H11	Patients who feel discriminated against or isolated because of their diet have lower adherence.
	**Economic and Accessibility Factors**
H12	Perceiving GF products as expensive is associated with lower dietary adherence.
H13	Greater availability of GF products in stores and restaurants is positively correlated with dietary adherence.
H14	Patients who receive nutritional education from specialists have higher adherence with diet therapy than those who self-educate.

**Table 4:** Auxiliary analyses and study questions.

№	Auxiliary Analyses and Study Questions
1	What is the overall rate of self-diagnosis among patients with GRDs?
2	Which patient group is more prone to self-diagnosis? (We initially hypothesized that those with NCGS would be more prone, as observed in other studies.)
3	Do GRDs show a hereditary component, such that individuals with CD report more affected relatives than those with NCGS?
4	Are patients with CD more likely to bring their own food to social events than those with NCGS?
5	Which patient group checks food labels more frequently—patients with CD or NCGS?
6	How frequently do individuals with GRDs, adhering to a GFD, experience symptoms after consuming products labeled as certified GF?
7	Is there an association between weight and GFD adherence, and between weight and time since diagnosis?
8	Which group finds the GFD to be more challenging—patients diagnosed with CD or those with NCGS?
9	Do those who feel discriminated against tend to isolate themselves more? Who isolate themselves more—patients with CD or NCGS?
10	Which group perceives the cost of dietary therapy as being higher—patients with CD or NCGS?
11	How does time impact GF food perceived availability in stores?
12	Is there a correlation between the duration since diagnosis and the perceived availability of GF food?
13	From whom do the patients tend to learn more about their diet?
14	Do those with NCGS tend to learn more about the diet from non-specialists?
15	Do patients who receive nutritional education from specialists perceive diet therapy as easier to follow than those who self-educate?
16	Are patients who receive nutritional education from specialists less prone to making unsuitable dietary choices for the GFD than those who self-educate?
17	How prevalent is LI among GRD patients?
18	What are the most commonly associated chronic diseases among GRD patients?
19	Among those who self-diagnosed, what are the most commonly associated chronic conditions?

**Table 5:** Descriptive statistics pertaining to GFD by gender.

Gender	*n*	Missing	Mean	SD	Min.	Max.
Male	107	0	6.673	0.822	3	7
Female	574	0	6.427	1.085	2	7

**Table 6:** Distribution of CD and NCGS in the sample.

Diagnosis	Frequency	Percentage
CD	539	79.148
NCGS	142	20.852
Total	681	100.000

**Table 7:** Distribution of participants by type of received diagnosis.

Diagnosed by	Frequency	Percentage
Specialist doctor	606	88.987
Self-diagnosis	75	11.013
Total	681	100.000

**Table 8:** Overall regression model significance for medical and individual factors.

Model	SS	*df*	MS	*F*	*p*
Regression	434.540	8	54.317	18.625	<0.001
Residual	1538.200	672	2.051		
Total	1972.740	680	Total		

**Table 9:** Regression coefficients for medical and individual factors.

Variable	Coefficient	Std. Err.	*t*	*p*
Family history of GRDs (answer: no)	0.093	0.141	0.660	0.509
Diagnosis (answer: CD)	1.167	0.169	9.748	<0.001
Time since diagnosis (answer: 1–2 yrs)	0.201	0.199	1.011	0.312
Time since diagnosis (answer: 2–5 yrs)	0.149	0.183	0.811	0.418
Time since diagnosis (answer: 5–10 yrs)	0.273	0.182	1.498	0.135
Time since diagnosis (answer: >10 yrs)	0.126	0.202	0.623	0.533
Self-diagnosed	−0.603	0.217	−2.783	0.006
Other chronic conditions (answer: no)	0.084	0.124	0.675	0.500

**Table 10:** Overall regression model significance for psychosocial factors.

Model	SS	*df*	MS	*F*	*p*
Regression	661.556	20	33.078	16.650	<0.001
Residual	1311.184	660	1.987		
Total	1972.740	680			

**Table 11:** Statistically significant regression coefficients for psychosocial factors.

Variable	Coefficient	Std. Err.	*t*	*p*
Perceived importance at diagnosis time (a little important)	−1.850	0.657	−2.817	0.005
Perceived importance now (moderately important)	−3.341	1.457	−2.294	0.022
Perceived importance now (important)	−3.022	1.432	−2.110	0.035
Absence from social contexts (sometimes)	0.452	0.174	2.594	0.010
Absence from social contexts (most times)	0.815	0.182	4.490	<0.001

**Table 12:** Overall regression model significance for economic factors and availability.

Model	Sum of Squares	*df*	Mean Square	*F*	*p*
Regression	172.570	21	8.218	3.008	<0.001
Residual	1800.170	659	2.732		
Total	1972.740	680			

**Table 13:** Statistically significant regression coefficients for economic factors and availability.

Variable	Coefficient	Std. Err.	*t*	*p*
GFD cost (moderately expensive)	−2.790	1.286	−2.168	0.030
GF food alternatives in restaurants (sometimes)	−0.688	0.231	−2.983	0.003
GF food alternatives in restaurants (always)	−2.325	0.990	−2.349	0.018

**Table 14:** Sources of information about the GFD.

Sources of Information About GFD	From a Specialist Doctor	From a Specialist Nutritionist- Dietitian	Self-Sourced Information	With the Help of Other People with the Same Diagnosis	Total
CD	22.82% (*n* = 123)	5.57% (*n* = 30)	48.98% (*n* = 264)	22.63% (*n* = 122)	100% (*n* = 539)
NCGS	12.68% (*n* = 18)	9.15% (*n* = 13)	63.38% (*n* = 90)	14.79% (*n* = 21)	100% (*n* = 142)
Overall	20.71% (*n* = 141)	6.31% (*n* = 43)	51.98% (*n* = 354)	21.00% (*n* = 143)	100% (*n* = 681)

## Data Availability

The original contributions presented in this study are included in the article/Supplementary Material. Further inquiries can be directed to the corresponding authors.

## Supplementary Materials

The following supporting information can be downloaded at: https://www.mdpi.com/article/10.3390/nu17233664/s1. Questionnaire S1; Table S1: Reported chronic disorders among patients with GRDs.

## Abbreviations

The following abbreviations are used in this manuscript:

ARIG	Romanian Association for Gluten Intolerance
CD	Celiac Disease
GC	Gluten-Containing
GERD	Gastroesophageal Reflux Disease
GF	Gluten-Free
GFD	Gluten-Free Diet
GRD	Gluten-Related Disorder
IBS	Irritable Bowel Syndrome
LI	Lactose Intolerance
NCGS	Non-Celiac Gluten Sensitivity
NCWS	Non-Celiac Wheat Sensitivity
QoL	Quality of Life
SRGS	Self-Reported Gluten Sensitivity

## References

[1] Jansson-Knodell C.L., Rubio-Tapia A. (2024). Gluten-Related Disorders from Bench to Bedside. Clin. Gastroenterol. Hepatol..

[2] Valenti S., Corica D., Ricciardi L., Romano C. (2017). Gluten-Related Disorders: Certainties, Questions and Doubts. Ann. Med..

[3] Al-Toma A., Volta U., Auricchio R., Castillejo G., Sanders D.S., Cellier C., Mulder C.J., Lundin K.E.A. (2019). European Society for the Study of Coeliac Disease (ESsCD) Guideline for Coeliac Disease and Other Gluten-Related Disorders. United Eur. Gastroenterol. J..

[4] Singh P., Arora A., Strand T.A., Leffler D.A., Catassi C., Green P.H., Kelly C.P., Ahuja V., Makharia G.K. (2018). Global Prevalence of Celiac Disease: Systematic Review and Meta-Analysis. Clin. Gastroenterol. Hepatol..

[5] Pinto-Sanchez M.I., Silvester J.A., Lebwohl B., Leffler D.A., Anderson R.P., Therrien A., Kelly C.P., Verdu E.F. (2021). Society for the Study of Celiac Disease Position Statement on Gaps and Opportunities in Coeliac Disease. Nat. Rev. Gastroenterol. Hepatol..

[6] Bonner E.R., Tschollar W., Anderson R. (2025). Review Article: Novel Enzyme Therapy Design for Gluten Peptide Digestion Through Exopeptidase Supplementation. Aliment. Pharmacol. Ther..

[7] Alkalay M.J. (2022). Nutrition in Patients with Lactose Malabsorption, Celiac Disease, and Related Disorders. Nutrients.

[8] Gnodi E., Meneveri R., Barisani D. (2022). Celiac Disease: From Genetics to Epigenetics. World J. Gastroenterol..

[9] Durazzo M., Ferro A., Brascugli I., Mattivi S., Fagoonee S., Pellicano R. (2022). Extra-Intestinal Manifestations of Celiac Disease. What Should We Know in 2022?. J. Clin. Med..

[10] Tomer R., Patiyal S., Dhall A., Raghava G.P.S. (2023). Prediction of Celiac Disease Associated Epitopes and Motifs in a Protein. Front. Immunol..

[11] Verdelli A., Corrà A., Mariotti E.B., Aimo C., Quintarelli L., di Calabria V.R., Donati M.E., Bonciolini V., Antiga E., Caproni M. (2023). Skin Gluten-Related Disorders: New and Old Cutaneous Manifestations to Be Considered. Front. Med..

[12] Yu X.B., Uhde M., Green P.H., Alaedini A. (2018). Autoantibodies in the Extraintestinal Manifestations of Celiac Disease. Nutrients.

[13] Pavelescu L.A., Sabau I.D., Sanda-Dira G., Iacata A.A., Curici A. (2025). Serological, Genetic, and Bi-ochemical Insights into Celiac Disease Diagnosis and Vitamin D Deficiency in Romanian Children: A Com-prehensive Cohort Study. Int. J. Mol. Sci..

[14] King J.A., Jeong J., Underwood F.E., Quan J., Panaccione N., Windsor J.W., Coward S., Debruyn J., Ronksley P.E., Shaheen A.A. (2020). Incidence of Celiac Disease Is Increasing over Time: A Systematic Review and Meta-Analysis. Am. J. Gastroenterol..

[15] Pop A., Popa S.L., Pop D.D., Ismaiel A., Rusu F., Grad S., Dumitrascu D.L. (2024). Prevalence of Celi-ac Disease in Romania. J. Gastrointest. Liver Dis..

[16] Al-Toma A., Zingone F., Branchi F., Schiepatti A., Malamut G., Canova C., Rosato I., Ocagli H., Trott N., Elli L. (2025). European Society for the Study of Coeliac Disease 2025 Updated Guidelines on the Diagnosis and Management of Coeliac Disease in Adults. Part 1: Diagnostic Approach. United Eur. Gastroen-Terology J..

[17] Rouvroye M.D., Oldenburg L., Slottje P., Joosten J.H.K., de Menezes R.X., Reinders M.E., Bouma G. (2021). Testing for Coeliac Disease Rarely Leads to a Diagnosis: A Population-Based Study. Scand. J. Prim. Health Care.

[18] Wagh S.K., Lammers K.M., Padul M.V., Rodriguez-Herrera A., Dodero V.I. (2022). Celiac Disease and Possible Dietary Interventions: From Enzymes and Probiotics to Postbiotics and Viruses. Int. J. Mol. Sci..

[19] Rahimi S., Mahmoudi Ghehsareh M., Asri N., Azizmohammad Looha M., Jahani-Sherafat S., Ciacci C., Ros-tami-Nejad M. (2025). Gluten-free diet adherence patterns and health outcomes in celiac disease: A retrospective observational study. BMC Gastroenterol..

[20] Stanciu D., Staykov H., Dragomanova S., Tancheva L., Pop R.S., Ielciu I., Crișan G. (2024). Gluten Unraveled: Latest Insights on Terminology, Diagnosis, Pathophysiology, Dietary Strategies, and Intestinal Microbiota Modulations—A Decade in Review. Nutrients.

[21] Pop A.V., Popa S.L., Dumitrascu D.L. (2024). Extra-digestive manifestations of celiac disease. Med. Pharm. Rep..

[22] Balaban V., Popp A., Vasilescu F., Ene A., Jinga M. (2016). Celiac Disease Phenotype in Clinically Diagnosed Romanian Adults and Children. Maedica.

[23] Goel G., Tye-Din J.A., Qiao S.W., Russell A.K., Mayassi T., Ciszewski C., Sarna V.K., Wang S., Goldstein K.E., Dzuris J.L. (2019). Cytokine Release and Gastrointestinal Symptoms after Gluten Challenge in Celiac Disease. Sci. Adv..

[24] Caio G., Volta U., Sapone A., Leffler D.A., De Giorgio R., Catassi C., Fasano A. (2019). Celiac Disease: A Comprehensive Current Review. BMC Med..

[25] Leffler D.A., Green P.H.R., Fasano A. (2015). Extraintestinal Manifestations of Coeliac Disease. Nat. Rev. Gastroenterol. Hepatol..

[26] Therrien A., Kelly C.P., Silvester J.A. (2020). Celiac Disease: Extraintestinal Manifestations and Associated Conditions. J. Clin. Gastroenterol..

[27] Nardecchia S., Auricchio R., Discepolo V., Troncone R. (2019). Extra-Intestinal Manifestations of Coeliac Disease in Children: Clinical Features and Mechanisms. Front. Pediatr..

[28] Singh A.D., Ellias S., Singh P., Ahuja V., Makharia G.K. (2022). The Prevalence of the Celiac Disease in Patients with Dyspepsia: A Systematic Review and Meta-Analysis. Dig. Dis. Sci..

[29] Lamjadli S., Oujamaa I., Souli I., Eddehbi F., Lakhouaja N., Bouchra M., Salami A., Guennouni M. (2025). Micronutrient Deficiencies in Patients with Celiac Disease: A Systematic Review and Meta-Analysis. Int. J. Immunopathol. Pharmacol..

[30] Lauret E., Rodrigo L. (2013). Celiac Disease and Autoimmune-Associated Conditions. Biomed. Res. Int..

[31] Schnedl W.J., Mangge H., Schenk M., Enko D. (2021). Non-Responsive Celiac Disease May Coincide with Additional Food Intolerance/Malabsorption, Including Histamine Intolerance. Med. Hypotheses.

[32] Dieterich W., Zopf Y. (2019). Gluten and FODMAPS—Sense of a Restriction/When. Nutrients.

[33] An C., Yang J., Pinto-Sanchez M.I., Verdu E.F., Lebwohl B., Green P.H., Alaedini A. (2025). Molecular Triggers of Non-Celiac Wheat Sensitivity: A Scoping Review and Analysis. Am. J. Gastroenterol..

[34] Elli L., Branchi F., Tomba C., Villalta D., Norsa L., Ferretti F., Roncoroni L., Bardella M.T. (2015). Diagnosis of Gluten Related Disorders: Celiac Disease, Wheat Allergy and Non-Celiac Gluten Sensitivity. World J. Gastroenterol..

[35] Manza F., Lungaro L., Costanzini A., Caputo F., Carroccio A., Mansueto P., Seidita A., Raju S.A., Volta U., De Giorgio R. (2025). Non-Celiac Gluten/Wheat Sensitivity—State of the Art: A Five-Year Narrative Review. Nutrients.

[36] Shahbazkhani B., Fanaeian M.M., Farahvash M.J., Aletaha N., Alborzi F., Elli L., Shahbazkhani A., Zebardast J., Rostami-Nejad M. (2020). Prevalence of Non-Celiac Gluten Sensitivity in Patients with Refractory Functional Dyspepsia: A Randomized Double-Blind Placebo Controlled Trial. Sci. Rep..

[37] Catassi C., Catassi G., Naspi L. (2023). Nonceliac Gluten Sensitivity. Curr. Opin. Clin. Nutr. Metab. Care.

[38] Expósito Miranda M., García-Valdés L., Espigares-Rodríguez E., Leno-Durán E., Requena P. (2023). Non-Celiac Gluten Sensitivity: Clinical Presentation, Etiology and Differential Diagnosis. Gastroenterol. Y Hepatol..

[39] Roszkowska A., Pawlicka M., Mroczek A., Bałabuszek K., Nieradko-Iwanicka B. (2019). Non-Celiac Gluten Sensitivity: A Review. Medicina.

[40] Graziano M., Rossi M. (2018). An Update on the Cutaneous Manifestations of Coeliac Disease and Non-Coeliac Gluten Sensitivity. Int. Rev. Immunol..

[41] Skodje G.I., Minelle I.H., Rolfsen K.L., Iacovou M., Lundin K.E.A., Veierød M.B., Henriksen C. (2019). Dietary and Symptom Assessment in Adults with Self-Reported Non-Coeliac Gluten Sensitivity. Clin. Nutr. ESPEN.

[42] Molina-Infante J., Santolaria S., Sanders D.S., Fernández-Bañares F. (2015). Systematic Review: Noncoeliac Gluten Sensitivity. Aliment. Pharmacol. Ther..

[43] Di Sabatino A., Volta U., Salvatore C., Biancheri P., Caio G., De Giorgio R., Di Stefano M., Corazza G.R. (2015). Small Amounts of Gluten in Subjects with Suspected Nonceliac Gluten Sensitivity: A Randomized, Double-Blind, Placebo-Controlled, Cross-Over Trial. Clin. Gastroenterol. Hepatol..

[44] Barbaro M.R., Cremon C., Stanghellini V., Barbara G. (2018). Recent Advances in Understanding Non-Celiac Gluten Sensitivity. F1000Research.

[45] Scalvini D., Scarcella C., Mantica G., Bartolotta E., Maimaris S., Fazzino E., Biagi F., Schiepatti A. (2025). Beyond gluten-free diet: A critical perspective on phase 2 trials on non-dietary pharmacological therapies for coeliac disease. Front. Nutr..

[46] Pultz I.S., Hill M., Vitanza J.M., Wolf C., Saaby L., Liu T., Winkle P., Leffler D.A. (2021). Gluten Degradation, Pharmacokinetics, Safety, and Tolerability of TAK-062, an Engineered Enzyme to Treat Celiac Disease. Gastroenterology.

[47] Syage J.A., Murray J.A., Green P.H.R., Khosla C. (2017). Latiglutenase Improves Symptoms in Seropositive Celiac Disease Patients While on a Gluten-Free Diet. Dig. Dis. Sci..

[48] Pinier M., Fuhrmann G., Galipeau H.J., Rivard N., Murray J.A., David C.S., Drasarova H., Tuckova L., Leroux J., Verdu E.F. (2012). The copolymer P(HEMA-co-SS) binds gluten and reduces immune response in gluten-sensitized mice and human tissues. Gastroenterology.

[49] Sample D.A., Sunwoo H.H., Huynh H.Q., Rylance H.L., Robert C.L., Xu B.W., Kang S.H., Gujral N., Dieleman L.A. (2017). AGY, a Novel Egg Yolk-Derived Anti-gliadin Antibody, Is Safe for Patients with Celiac Disease. Dig. Dis. Sci..

[50] Stadlmann V., Harant H., Korschineck I., Hermann M., Forster F., Missbichler A. (2015). Novel avian single-chain fragment variable (scFv) targets dietary gluten and related natural grain prolamins, toxic entities of celiac disease. BMC Biotechnol..

[51] Leffler D.A., Kelly C.P., Green P.H.R., Fedorak R.N., Dimarino A., Perrow W., Rasmussen H., Wang C., Bercik P., Bachir N.M. (2015). Larazotide acetate for persistent symptoms of celiac disease despite a gluten-free diet: A randomized controlled trial. Gastroenterology.

[52] Attarwala H., Clausen V., Chaturvedi P., Amiji M.M. (2017). Cosilencing Intestinal Transglutaminase-2 and Interleukin-15 Using Gelatin-Based Nanoparticles in an In Vitro Model of Celiac Disease. Mol. Pharm..

[53] Schuppan D., Mäki M., Lundin K.E.A., Isola J., Friesing-Sosnik T., Taavela J., Popp A., Koskenpato J., Langhorst J., Hovde Ø. (2021). A Randomized Trial of a Transglutaminase 2 Inhibitor for Celiac Disease. N. Engl. J. Med..

[54] Buriánek F., Gege C., Marinković P. (2024). New Developments in Celiac Disease Treatments. Drug Discov. Today.

[55] Hardy M.Y., Henneken L.M., Russell A.K., Okura Y., Mizoroki A., Ozono Y., Kobayashi S., Murakami Y., Tye-Din J.A. (2024). A bispecific antibody targeting HLA-DQ2.5-gluten peptides potently blocks gluten-specific T cells induced by gluten ingestion in patients with celiac disease. Clin. Immunol..

[56] Kelly C.P., Murray J.A., Leffler D.A., Getts D.R., Bledsoe A.C., Smithson G., First M.R., Morris A., Boyne M., Elhofy A. (2021). TAK-101 Nanoparticles Induce Gluten-Specific Tolerance in Celiac Disease: A Randomized, Double-Blind, Placebo-Controlled Study. Gastroenterology.

[57] Murray J.A., Wassaf D., Dunn K., Arora S., Winkle P., Stacey H., Cooper S., Goldstein K.E., Manchanda R., Kontos S. (2023). Safety and tolerability of KAN-101, a liver-targeted immune tolerance therapy, in patients with coeliac disease (ACeD): A phase 1 trial. Lancet Gastroenterol. Hepatol..

[58] Theron M., Bentley D., Nagel S., Manchester M., Gerg M., Schindler T., Silva A., Ecabert B., Teixeira P., Perret C. (2017). Pharmacodynamic monitoring of RO5459072, a small molecule inhibitor of cathepsin S. Front. Immunol..

[59] Yoosuf S., Makharia G.K. (2022). Treatment of Gluten-Related Disorders. Gluten-Related Disorders: Diagnostic Approaches, Treatment Pathways, and Future Perspectives.

[60] Cohen J. (1988). Statistical Power Analysis for the Behavioral Sciences.

[61] Al-sunaid F.F., Al-homidi M.M., Al-qahtani R.M., Al-ashwal R.A., Mudhish G.A., Hanbazaza M.A., Al-zaben A.S. (2021). The Influence of a Gluten-Free Diet on Health-Related Quality of Life in Individuals with Celiac Disease. BMC Gastroenterol..

[62] Demirkesen I., Ozkaya B. (2022). Recent Strategies for Tackling the Problems in Gluten-Free Diet and Products. Crit. Rev. Food Sci. Nutr..

[63] Bascuñán K.A., Vespa M.C., Araya M. (2017). Celiac Disease: Understanding the Gluten-Free Diet. Eur. J. Nutr..

[64] Green P.H.R., Lebwohl B., Greywoode R. (2015). Celiac Disease. J. Allergy Clin. Immunol..

[65] Cheng F.W., Handu D. (2020). Nutrition Assessment, Interventions, and Monitoring for Patients with Celiac Disease: An Evidence Analysis Center Scoping Review. J. Acad. Nutr. Diet..

[66] Cabrera-Chávez F., Dezar G.V.A., Islas-Zamorano A.P., Espinoza-Alderete J.G., Vergara-Jiménez M.J., Magaña-Ordorica D., Ontiveros N. (2017). Prevalence of Self-Reported Gluten Sensitivity and Adherence to a Gluten-Free Diet in Argentinian Adult Population. Nutrients.

[67] Niland B., Cash B.D. (2018). Health Benefits and Adverse Effects of a Gluten-Free Diet in Non-Celiac Disease Patients. Gastroenterol. Hepatol..

[68] Volta U., Bardella M.T., Calabrò A., Troncone R., Corazza G.R., Bagnato C., Belcari C., Bellantoni A., Caio G., Calella F. (2014). An Italian Prospective Multicenter Survey on Patients Suspected of Having Non-Celiac Gluten Sensitivity. BMC Med..

[69] van Gils T., Nijeboer P., Ijssennagger C.E., Sanders D.S., Mulder C.J.J., Bouma G. (2016). Prevalence and Characterization of Self-Reported Gluten Sensitivity in The Netherlands. Nutrients.

[70] Carroccio A., Giambalvo O., La Blasca F., Iacobucci R., D’Alcamo A., Mansueto P. (2017). Self-Reported Non-Celiac Wheat Sensitivity in High School Students: Demographic and Clinical Characteristics. Nutrients.

[71] Zanini B., Baschè R., Ferraresi A., Ricci C., Lanzarotto F., Marullo M., Villanacci V., Hidalgo A., Lanzini A. (2015). Randomised Clinical Study: Gluten Challenge Induces Symptom Recurrence in Only a Minority of Patients Who Meet Clinical Criteria for Non-Coeliac Gluten Sensitivity. Aliment. Pharmacol. Ther..

[72] Herfindal A.M., Nilsen M., Aspholm T.E., Schultz G.I.G., Valeur J., Rudi K., Thoresen M., Lundin K.E.A., Henriksen C., Bøhn S.K. (2024). Effects of fructan and gluten on gut microbiota in individuals with self-reported non-celiac gluten/wheat sensitivity—A randomised controlled crossover trial. BMC Med..

[73] Jansson-Knodell C.L., White M., Lockett C., Xu H., Rubio-Tapia A., Shin A. (2023). Self-Reported Gluten Intolerance Is Prevalent, but Not All Gluten-Containing Foods Are Equal. Dig. Dis. Sci..

[74] Schiepatti A., Savioli J., Vernero M., de Andreis F.B., Perfetti L., Meriggi A., Biagi F. (2020). Pitfalls in the Diagnosis of Coeliac Disease and Gluten-Related Disorders. Nutrients.

[75] Silvester J.A., Weiten D., Graff L.A., Walker J.R., Duerksen D.R. (2016). Living Gluten-Free: Adherence, Knowledge, Lifestyle Adaptations and Feelings towards a Gluten-Free Diet. J. Human. Nutr. Diet..

[76] Jeanes Y., Orlandi L., Muhammad H., Reeves S. (2025). Telemedicine in Coeliac Disease: In-Person Appointments Are Favoured by Patients With a Lower Education Attainment and Lower Household Income. J. Human. Nutr. Diet..

[77] Centrul Regional Pentru Managementul Bolii Celiace. https://www.insmc.ro/compartimente/centrul-regional-pentru-managementul-bolii-celiace/.

[78] Siminiuc R., Ţurcanu D. (2022). Food Security of People with Celiac Disease in the Republic of Moldova through Prism of Public Policies. Front. Public Health.

[79] Members of Association of European Coeliac Societies|AOECS. https://www.aoecs.org/about-us/members/.

[80] Asociația Română Pentru Intoleranță La Gluten (ARIG). https://arig.ro/organizare/.

[81] Avena-Woods C., Mangione R.A., Wu W.K. (2018). Exploring the Community Pharmacist’s Knowledge of Celiac Disease. Am. J. Pharm. Educ..

[82] Poslt Königová M., Sebalo Vňuková M., Řehořková P., Anders M., Ptáček R. (2023). The Effectiveness of Gluten-Free Dietary Interventions: A Systematic Review. Front. Psychol..

[83] Isaac D.M., Wu J., Mager D.R., Turner J.M. (2016). Managing the Pediatric Patient with Celiac Disease: A Multidisciplinary Approach. J. Multidiscip. Healthc..

[84] Kaul R., Jansson-Knodell C., Simons M.L., Weekley K., Gardinier D., Rubio-Tapia A. (2025). Avoidant/Restrictive Food Intake Disorder in Celiac Disease. Nutrients.

[85] Zarkadas M., Dubois S., Macisaac K., Cantin I., Rashid M., Roberts K.C., La Vieille S., Godefroy S., Pulido O.M. (2013). Living with Coeliac Disease and a Gluten-Free Diet: A Canadian Perspective. J. Human Nutr. Diet..

[86] Leffler D.A., Edwards-George J., Dennis M., Schuppan D., Cook F., Franko D.L., Blom-Hoffman J., Kelly C.P. (2008). Factors That Influence Adherence to a Gluten-Free Diet in Adults with Celiac Disease. Dig. Dis. Sci..

[87] Lebwohl B., Sanders D.S., Green P.H.R. (2018). Coeliac Disease. Lancet.

[88] Husby S., Koletzko S., Korponay-Szabó I.R., Mearin M.L., Phillips A., Shamir R., Troncone R., Giersiepen K., Branski D., Catassi C. (2012). European Society for Pediatric Gastroenterology, Hepatology, and Nutrition Guidelines for the Diagnosis of Coeliac Disease. J. Pediatr. Gastroenterol. Nutr..

[89] Burden M., Mooney P.D., Blanshard R.J., White W.L., Cambray-Deakin D.R., Sanders D.S. (2015). Cost and Availability of Gluten-Free Food in the UK: In Store and Online. Postgrad. Med. J..

[90] Ribeiro C.d.S., Pratesi C.B., Zandonadi R.P. (2025). Celiac Disease and Gluten-Free Diets: A Path or Barrier to Food (In) Security?. Nutrients.

[91] Rubio-Tapia A., Hill I.D., Kelly C.P., Calderwood A.H., Murray J.A. (2013). ACG Clinical Guidelines: Diagnosis and Management of Celiac Disease. Am. J. Gastroenterol..

[92] Ludvigsson J.F., Murray J.A. (2019). Epidemiology of Celiac Disease. Gastroenterol. Clin. N. Am..

[93] Bardella M.T., Fredella C., Saladino V., Trovato C., Cesana B.M., Quatrini M., Prampolini L. (2005). Gluten Intolerance: Gender-and Age-Related Differences in Symptoms. Scand. J. Gastroenterol..

[94] Jansson-Knodell C.L., Hujoel I.A., West C.P., Taneja V., Prokop L.J., Rubio-Tapia A., Murray J.A. (2019). Sex Difference in Celiac Disease in Undiagnosed Populations: A Systematic Review and Meta-Analysis. Clin. Gastroenterol. Hepatol..

[95] Ciccocioppo R., Kruzliak P., Cangemi G.C., Pohanka M., Betti E., Lauret E., Rodrigo L. (2015). The Spectrum of Differences between Childhood and Adulthood Celiac Disease. Nutrients.

[96] Megiorni F., Mora B., Bonamico M., Barbato M., Montuori M., Viola F., Trabace S., Mazzilli M.C. (2008). HLA-DQ and Susceptibility to Celiac Disease: Evidence for Gender Differences and Parent-of-Origin Effects. Am. J. Gastroenterol..

[97] Hernangomez-Laderas A., Cilleros-Portet A., Martínez Velasco S., Marí S., Legarda M., González-García B.P., Tutau C., García-Santisteban I., Irastorza I., Fernandez-Jimenez N. (2023). Sex Bias in Celiac Disease: XWAS and Monocyte EQTLs in Women Identify TMEM187 as a Functional Candidate Gene. Biol. Sex. Differ..

[98] Klein S.L., Flanagan K.L. (2016). Sex Differences in Immune Responses. Nat. Rev. Immunol..

[99] Salazar C., García-Cárdenas J.M., Paz-y-miño C. (2017). Understanding Celiac Disease from Genetics to the Future Diagnostic Strategies. Clin. Med. Insights Gastroenterol..

[100] Dochat C., Afari N., Satherley R.-M., Coburn S., McBeth J.F. (2024). Celiac Disease Symptom Profiles and Their Relationship to Gluten-Free Diet Adherence, Mental Health, and Quality of Life. BMC Gastroenterol..

[101] Sapone A., Bai J.C., Ciacci C., Dolinsek J., Green P.H.R., Hadjivassiliou M., Kaukinen K., Rostami K., Sanders D.S., Schumann M. (2012). Spectrum of Gluten-Related Disorders: Consensus on New Nomenclature and Classification. BMC Med..

