# Supplementary material for: Living Gluten-Free in Romania: A National Cross-Sectional Study of Dietary Adherence in Clinically Diagnosed and Self-Reported Cases
Source: Nutrients. 2025 Nov 23;17(23):3664. doi: 10.3390/nu17233664 (PMC12694147; doi:10.3390/nu17233664)
Supplement: Supplementary file 1 [file nutrients-17-03664-s001.zip › Supplementary Questionnaire S1.pdf]

Supplementary Questionnaire S1

Introductory text "This study is conducted within the [*institution name*] and aims to analyze the gluten intolerance (celiac/non-celiac) among patients with Romanian citizenship, as well as diet therapy and patient adherence.

Participation in this research is not associated with any medical risk or benefit for you and only involves completing this online questionnaire.

The answers provided are anonymous.

The answers of minors will be provided by legal guardians, so that legal guardians can complete the questionnaire for the minors suffering from these conditions.

With * you find the questions that you must answer.

Thank you for your involvement!"

Agreement to participate in the study

Before starting to fill out the questionnaire, we inform you that the data provided will be processed for statistical and scientific purposes only. The inclusion criteria for the study subjects will include the possibility of providing their consent to be included in the study and completing a questionnaire; the presence of a pathology associated with gluten consumption; Romanian citizenship. The exclusion criteria for the study subjects will include citizenship other than Romanian, the absence of a pathology associated with gluten consumption, refusal to provide consent to be included in the study. We assure you that the necessary measures have been taken to protect and ensure the confidentiality of the data and we will prevent the breach of the security of personal information, in accordance with the legislation in force. Below is the inclusion agreement for the study, which allows participation in it.

1. The undersigned, as a subject investigated in the project entitled [*project name*], having as principal investigator [*investigator name*], I declare that I agree that [*the investigator*] may process the data provided by me anonymously in the present study (respectively the answers to the questions). My consent is given voluntarily in order to participate in the present study. I declare that I meet the inclusion criteria in the study and that I do not align myself with the exclusion criteria. I have understood this declaration of consent, and I agree to the processing of the data provided by me through this questionnaire, for the purposes described herein. *
   Yes, I agree
   I do not agree

The questionnaire

General information

2. I live in *
   Romania
   Other country ___________ (state the country here)

3. My gender is *
   M
   F
   I do not want to answer

4. I was diagnosed with * (multiple answers possible)
   Non-celiac gluten intolerance (sensitivity)
   Celiac gluten intolerance (Celiac disease)
   Lactose intolerance

5. I was diagnosed by *
   The specialist doctor
   Self-diagnosis (I excluded some food categories on my own initiative and found significant improvements in symptoms)

6. I was diagnosed *
   Less than 1 year ago
   1-2 years ago
   2-5 years ago
   5-10 years ago
   More than 10 years ago

7. In my family there are other people (siblings, parents, grandparents etc.) who have my condition related to gluten consumption. *
   Yes
   No

8. If Yes, how many people and what is the degree of relationship?
   ___________________ (write the numbers here and the relationship degree)

9. For me, accepting the diagnosis was *
   Not difficult
   Moderately difficult
   Very difficult
   Extremely difficult

10. In addition to the previously mentioned diagnosis(es), I also present other chronic diseases (e.g. high blood pressure, diabetes etc.). *
    Yes
    No

11. If Yes, these diseases are:
    ___________________ (here the diseases are written)

The diet I follow related to my diagnosis

12. After my diagnosis, I learned about the gluten-free diet that I have to follow mostly *
    From a specialist doctor
    From a specialist nutritionist-dietitian
    On my own
    With the help of other people with the same diagnosis

13. At first (immediately after receiving the diagnosis), I considered the gluten-free diet to be*
    Very important
    Important
    Moderately important
    A little important
    Not important

14. Now, I consider the gluten-free diet to be*
    Very important
    Important
    Moderately important
    A little important
    Not important

15. At first, I considered the gluten-free diet to be *
    Difficult
    Acceptable
    Easy

16. Now, I consider the gluten-free diet to be *
    Difficult
    Acceptable
    Easy

17. At first, I found food alternatives in stores (e.g. gluten-free bread etc.) *
    Always
    Often
    Sometimes
    Rarely
    Never

18. Now, I find food alternatives in stores (e.g. gluten-free bread etc.) *
    Always
    Often
    Sometimes
    Rarely
    Never

19. At first, I found food alternatives in restaurant menus (e.g. gluten-free pizza, gluten-free dessert etc.)*
    Always
    Often
    Sometimes
    Rarely
    Never

20. Now, I find food alternatives in restaurant menus (e.g. gluten-free pizza, gluten-free dessert etc.) *
    Always
    Often
    Sometimes
    Rarely
    Never

21. After diagnosis and starting the gluten-free diet *
    I lost weight
    I gained weight
    My weight stayed the same

22. Due to my diagnosis and the gluten-free diet I follow, I sometimes feel discriminated against by my family and friends. (e.g. When we meet up there are no food alternatives for me etc.) *
    True
    False

The socioeconomic impact

23. Due to my diagnosis and the gluten-free diet I follow, I have had to be absent from certain social contexts. (e.g. parties, dining out, family gatherings etc.) *
    Always
    In most situations (over 70% of these situations)
    Sometimes (in 40-60% of these situations)
    Rarely (in 10-30% of these situations)
    Never

24. I consider my special diet and gluten-free alternatives to be *
    Extremely expensive
    Very expensive
    Moderately expensive
    A little expensive
    Not expensive

My compliance and adherence to the recommended diet therapy

25. I follow the gluten-free diet *
    Always, without exception
    Most of the time
    Sometimes
    Rarely
    Never

26. There are situations in which I voluntarily consume gluten. *
    True
    False

27. I believe it is okay to sometimes eat foods that do not conform to my diet (foods containing gluten).*
    True
    False

28. I check the labels and packaging of the products I buy to make sure they are suitable for the gluten-free diet. *
    True
    False

29. I only consume foods certified as gluten-free. *
    True
    False

30. Although it is often difficult, I only eat foods that are suitable for a gluten-free diet. *
    True
    False

31. I ate foods that were certified gluten-free and then had symptoms that proved the certification was untrue. *
    True
    False

32. In social contexts where gluten-free foods are not served, I prefer not to eat anything or to bring foods that are suitable for my diet. *
    True
    False

33. In social contexts, I prefer not to tell anyone about my diet and to eat the foods that everyone present eats. *
    True
    False

Bonus Section – Testimonial

My story. How did I discover my condition? How has it changed my life? What do I wish I had known from the beginning, but had to learn the hard way along the way? What advice would I give to someone who has just been diagnosed? (this is optional, complete with text)
