# Supplementary material for: Living Gluten-Free in Romania: A National Cross-Sectional Study of Dietary Adherence in Clinically Diagnosed and Self-Reported Cases
Source: Nutrients. 2025 Nov 23;17(23):3664. doi: 10.3390/nu17233664 (PMC12694147; doi:10.3390/nu17233664)
Supplement: Supplementary file 1 [file nutrients-17-03664-s001.zip › Supplementary Table S1.pdf]

Supplementary Table S1.

Table S1. Reported chronic disorders among patients with GRDs.

Category of Disorders	Total (%)	Diagnosis	Total (%)	CD (%)	NCGS (%)
**Autoimmune**	56.3	Autoimmune Diseases (not specified)	0.9	0.4	0.4
		Autoimmune Gastritis	1.3	1.3	
		Autoimmune Hepatitis	0.4	0.4	
		Autoimmune Episcleritis	0.4	0.4	
		Autoimmune Thyroiditis (not specified)	18.6	13.4	5.2
		Basedow-Graves' Disease	1.3	0.9	0.4
		Hashimoto's Disease	12.1	9.5	2.6
		Ankylosing Spondylitis	0.9		0.9
		Vitiligo	1.7	1.3	0.4
		Psoriasis	2.2	1.7	0.4
		Lymphocytic Colitis	0.9	0.4	0.4
		Sjogren's Syndrome	3.0	2.6	0.4
		Rheumatoid Arthritis	2.2	1.7	0.4
		Dermatomyositis	0.4		0.4
		Polymyositis	0.4		0.4
		Type 1 Diabetes	5.6	5.6	
		Crohn's Disease	1.3	0.4	0.9
		Primary Biliary Cholangitis	0.9	0.9	
		Hidradenitis Suppurativa	0.4	0.4	
		Sicca Syndrome	0.4	0.4	
		Antiphospholipid Syndrome	0.4	0.4	
		Behçet's Disease	0.4	0.4	
**Neurological/ Psychiatric**	9.1	ADHD	0.4	0.4	
		Anxiety	0.9	0.4	0.4
		Panic Attacks	0.4		0.4
		Autism	0.4		0.4
		Epilepsy	1.3	1.3	
		Writer's Cramp	0.4	0.4	
		Chronic Fatigue	0.4	0.4	
		Mental/Brain Fog	0.4	0.4	
		Migraines	1.3	0.4	0.9
		Multiple Sclerosis	0.4	0.4	
		Benign Fasciculations	0.4	0.4	
		Neuropathy	1.7	1.3	0.4
		Spina Bifida	0.4	0.4	
**Cardiovascular/ Metabolic**	19.5	High Blood Pressure	14.7	9.5	5.2
		Paroxysmal Tachycardia	0.4		0.4
		Postural Tachycardia	0.4	0.4	
		Tachycardia	0.9		0.9
		Hypercholesterolemia	0.9	0.9	
		Dyslipidemia	0.4		0.4
		Metabolic Syndrome	0.4	0.4	
		Heart Failure	0.4	0.4	
		Syncope Upon Awakening	0.4	0.4	
		Extrasystoles	0.4	0.4	

Category of Disorders	Total (%)	Diagnosis	Total (%)	CD (%)	NCGS (%)
**Respiratory/ Allergic/ Immune**	20.3	Asthma	7.4	5.6	1.7
		Allergic Asthma	2.2	1.3	0.9
		Chronic Obstructive Pulmonary Disease	0.9		0.9
		Emphysema and Pulmonary Nodules	0.4	0.4	
		Respiratory allergies	0.4	0.4	
		Allergic Rhinitis	1.3	0.9	0.4
		Multiple Allergies (not specified)	3.0	0.9	2.2
		Pollen Allergies	0.4	0.4	
		Sinusitis	0.4	0.4	
		Food Intolerances (excluding lactose intolerance)	0.9		0.9
		Milk Protein Intolerance	0.4	0.4	
		Cow's Milk Protein Allergy	0.4		0.4
		Histamine Intolerance	2.2	1.3	0.9
**Gastrointestinal**	16.5	Gastritis	3.5	2.2	1.3
		Gastroduodenitis	0.4	0.4	
		Gastroesophageal Reflux Disease	2.2	0.9	1.3
		Duodenitis	0.4		0.4
		Gastric Hyperacidity	0.4	0.4	
		Esophagitis	0.4	0.4	
		Eosinophilic Colitis	0.4	0.4	
		Collagenous Microscopic Colitis	0.4	0.4	
		Irritable Bowel Syndrome	2.6		2.6
		Constipation	0.9	0.4	0.4
		Pancreatitis	0.4	0.4	
		Pancreatic Insufficiency	1.7	1.7	
		Pancreatic Cysts	0.4	0.4	
		Chronic Pelvic Inflammatory Disease	0.4	0.4	
		Hemorrhoids	0.4		0.4
		Hepatomegaly	0.9		0.9
		Hepatic Cytolysis	0.4	0.4	
**Endocrine**	18.2	Diabetes (not specified)	3.9	3.0	0.9
		Diabetes Insipidus	0.4	0.4	
		Prediabetes	2.6	1.3	1.3
		Thyroid Nodules	0.9	0.9	
		Nodular Goiter	0.4		0.4
		Thyroid Dysfunction (not specified)	0.9	0.4	0.4
		Thyroid Cyst	0.4		0.4
		Hyperparathyroidism	0.4	0.4	
		Hyperthyroidism	0.4	0.4	
		Hypothyroidism	7.4	4.3	3.0
		Pituitary Dwarfism	0.4	0.4	
**Musculoskeletal/ Connective Tissue**	9.1	Bone and Back Diseases (not specified)	0.4	0.4	
		Fibromyalgia	0.4	0.4	
		Cervical Hernia	0.9		0.9
		Gonarthrosis	0.4		0.4
		Osteoporosis	3.0	3.0	
		Osteopenia	0.4	0.4	
		Osteoarthritis	0.9	0.9	
		Spine Dysfunctions (not specified)	0.4	0.4	
		Strümpell-Lorrain Disease	0.9	0.9	

Category of Disorders	Total (%)	Diagnosis	Total (%)	CD (%)	NCGS (%)
		Chronic Arthritis	0.4	0.4	
		Polyarthritis	0.4	0.4	
		Lumbar Disc Herniation	0.4	0.4	
**Hematologic/ Genetic**	6.9	Lymphadenopathy	0.9	0.9	
		Thrombophilia	0.4	0.4	
		Anemia	2.2	2.2	
		Granulomatous Disease	0.4	0.4	
		Beta-Thalassemia Minor	0.9	0.9	
		Von Willebrand Disease	0.4	0.4	
		IgA Deficiency	0.4	0.4	
		Hypogammaglobulinemia	0.4	0.4	
		Immunodeficiency	0.4	0.4	
		Severe Neutropenia	0.4	0.4	
**Dermatologic**	3	Alopecia	0.9	0.9	
		Atopic Dermatitis	0.9	0.4	0.4
		Dermatitis Herpetiformis	0.4	0.4	
		Pityriasis Versicolor	0.4	0.4	
		Skin Diseases (not specified)	0.4	0.4	
**Gynecologic/ Reproductive**	4.3	Endometriosis	3.5	2.2	1.3
		Premature Ovarian Failure	0.4		0.4
		Infertility	0.4	0.4	
**Ophthalmologic**	1.3	Deep Amblyopia	0.4	0.4	
		Glaucoma	0.4	0.4	
		Ocular Hypertension	0.4	0.4	
**Renal/ Urologic**	1.3	Nephrotic Syndrome	0.4	0.4	
		Prostatitis	0.4		0.4
		Chronic Kidney Disease	0.4	0.4	
**Oncologic**	1.7	Neoplasia (not specified)	0.4		0.4
		Hodgkin Lymphoma	0.4	0.4	
		Uterine Neoplasia	0.4		0.4
		Thyroid Carcinoma	0.4		0.4
**Infectious/ Post-Infectious**	2.2	Lyme Disease	0.4	0.4	
		Hepatitis B/C/D	1.3	1.3	
		Hepatitis (not specified)	0.4	0.4	
**Not Specified**	2.6				
